# Assessing the rainfall infiltration on FOS via a new NSRM for a case study at high rock slope stability

**DOI:** 10.1038/s41598-022-15350-z

**Published:** 2022-07-13

**Authors:** Tianbai Zhou, Lingfan Zhang, Jian Cheng, Jianming Wang, Xiaoyu Zhang, Maoyuan Li

**Affiliations:** 1 Research Institute of Mine Big Data, China Coal Research Institute, Beijing, 100013 China; 2 State Key Laboratory of Coal Mining and Clean Utilization, Beijing, 100013 China; 3 Science and Technology Research Institute, China Three Gorges Corporation, Beijing, 100038 China; 4 School of Mechanics and Civil Engineering, China University of Mining and Technology, Beijing, 100083 China; 5 Ansteel Beijing Research Institute, Beijing, 102209 China; 6 Housing and Urban Rural Development Bureau, Qionglai City, Sichuan, 611500 China

**Keywords:** Natural hazards, Solid Earth sciences, Engineering

## Abstract

Assessing the stability characteristics of high rock slope under rainfall via theoretical research, numerical simulation, and field monitoring is of great implications for safety construction in open-pit mine engineering. Thus, based on the Hoke-Brown criterion, instantaneous internal friction angle and cohesion of high-slope rock mass under high stress conditions were deduced, and a nonlinear strength reduction method for high rock slope was established. The safety factors of the open-pit mine were calculated by COMSOL Multiphysics, which considering the high rock southwest slope and detected rainfall in Dagushan Open-pit Mine, China. The results showed that high rock slope stability could be more accurately analyzed by the proposed method due to its full consideration of slope stress state effect compared with the equivalent Mohr- Coulomb method. When the slope is low, the difference between the calculation results of the equivalent Mohr- Coulomb method and the proposed method is small, but with the increase of the slope height, the difference between the two calculation results gradually increases. When the transient saturated is formed in the slope surface layer and gradually increases, the reduction rate of the factor of safety (FOS) gradually increases. When the total rainfall is the same, the effect of short-term heavy rainfall on slope stability is less than that of long-term ordinary rainfall. The results obtained form this work provided important insights into the stability of high rock slope and rainfall infiltration in open-pit mine, and the safety factor is crucial for guiding the mining process design.

## Introduction

As China's “the belt and road” strategy is implemented, a new upsurge in construction of hydropower stations, mines, highways and railways in China is in the making. These projects will inevitably face high and steep slope problems. High rock slope has the characteristics of large scale, complex geological conditions and high geostress, and its stability is the key for the engineering construction. According to the statistics of landslides in open-pit mines in China in recent 30 years, there are more than ten landslides with large scale, which lead to serious economic and personnel losses. It is found that most of the large-scale landslides in these mines are directly related to the action of water, and most of them occur in the rainy season^1–4^. The direct reason is that the rainfall infiltration forms a transient saturation zone in the slope body, which reduces the safety factor. Therefore, the stability analysis of high rock slope under rainfall has become a key issue in the open-pit mine engineering^5,6^.

Rainfall has a greater influence on slope stability. The shear strength of the weak surface will be reduced by water immersion and infiltration, leading to an increase in sliding force, which will result in slope damage. Rainfall is the main factor that induces landslides on slopes, and the study of rainfall infiltration theory has been one of the research focuses in the field of slope engineering. Rainfall infiltration will cause changes in the seepage field of slope rock, which will change the stress field and eventually lead to slope instability and damage. The evolution of slope seepage field and slope seepage characteristics under rainfall infiltration are the basis for studying the impact of rainfall infiltration on slope stability^7,8^. Several studies have been conducted for rainfall infiltration-induced slope landslides. Rezaur et al.^9^ studied the influence of rainfall infiltration on the slope stability when the saturated permeability coefficient changes. Alonso et al.^10^ considered the influence of rainfall duration, rainfall intensity, soil type, shape of soil water characteristic curve and soil permeability on slope stability. Zhang et al.^11^ studied the influence of different water characteristic curve shapes on the pore-water pressure distribution of slope. Rahimi et al.^12^ studied the influence of water characteristic curve parameters on slope stability with different drainage effects.

In recent decades, with the rapid development of computer technology, numerical analysis method has been widely used in slope engineering^13–16^. On this basis, the strength reduction method has gradually become the main method to study the stability of rock slope. Compared with other methods, the rock stress–strain relationship could be considered by this method and that obtain the critical sliding surface and the minimum safety factor without assuming the shape and position of the sliding surface^17–20^. In the meantime, the progressive failure process of slope could be simulated by the strength reduction method. The generalized Hoek–Brown has been proved to be effective in the empirical calculation of shear strength of rock mass^21,22^. Therefore, it is necessary to combine the strength reduction method with Hoek–Brown criterion^23–25^. Many researchers have worked on the study and implementation of strength reduction method (SRM) with the nonlinear generalized Hoek–Brown (GHB) criterion, referred to as the nonlinear strength reduction method (NSRM)^26–29^, as shown in Table 1. Hammah et al.^30^ proposed a global GHB shear strength reduction method by lowering the GHB shear envelopes. Benz et al.^31^ established a mathematical expression for the relationship between the GHB criterion and the MC criterion, and used the MC criterion reduction factors to determine the strength reduction based on GHB criterion. Wu et al.^32^ concluded that for intact rock, only uniaxial compressive strength and rock hardness should be used as strength indicators for GHB strength reduction. Fu et al.^33^ introduced the Mohr–Coulomb instantaneous friction angle as a variable, used the instantaneous Moore-Coulomb friction angle and cohesive strength to describe the shear strength of rock mass, and proposed a nonlinear shear strength reduction technique for slope stability calculations. Sun et al.^34^ calculated the factor of safety (FOS) by combining slope geometric parameters, rock mass property parameters and GHB parameters, and proposed a simplified method to realize nonlinear strength reduction based on GHB criterion.

**Table 1:** The most important nonlinear strength reduction methods used in slope stability analysis.

Method	Characteristic	Application	References
Equivalent Mohr–Coulomb method	Equivalent strength parameter equation is solved for the equivalent cohesion and internal friction angle, and the safety factor is solved by the Mohr–Coulomb criterion	Basic nonlinear strength reduction method	Hoek and Brown^14^
Material parameters reduction method	Hoek–Brown material parameters m_b_, s are directly discounted	For Finite Element calculation of slope failure examples	Lin^28^
Global reduction of GHB envelopes method	Directly shear strength reduction by GHB	Combining GHB with SSR analysis for the Finite Element simulation	Hammah^30^
Local linearization method	Instantaneous internal friction angle and cohesion are solved by Newton's iterative method based on the relationship between normal stress and shear stress	Computing combined with FLAC^3D^	Lin^29^
Extended HB criterion reduction method	Strength reduction based on the extended HB criterion	For Finite Element calculation of slope failure examples	Benz^31^
Dual factoring strategy strength reduction method	Strength reduction based on uniaxial compressive strength and rock hardness	Numerical tests for a homogeneous, simple slope	Wu^32^
Nonlinear shear strength reduction technique	Introduced the Mohr–Coulomb instantaneous friction angle as a variable	A slope example is selected to verify the technique	Fu^15^

In summary, the nonlinear strength reduction methods have been proposed by previous researchers from different perspectives, but a strong focus on the slope stress state is missing. In practical engineering, especially in the application of high rock slope, the stress variation range is large, and the strength parameters of rock mass need to be reasonably determined for strength reduction. For high rock slope, the high stress state of rock mass is the most critical factor for strength reduction. Therefore, a nonlinear strength reduction method considering high stress state should be proposed for the FOS calculation. These knowledge gaps are seen as the main objectives in this work of stability analysis on the high rock slope. Aiming at the high stress of high rock slope, the instantaneous internal friction angle and instantaneous cohesion of rock mass under different stress states are deduced, and the new nonlinear strength reduction method for high rock slope is established according to the relationship between normal stress and shear stress of rock mass under the Hoke-Brown criterion. Taking the southwest slope of Dagushan Iron Mine as the research background, the safety factors of high rock slope under different rainfall conditions are calculated by the proposed nonlinear strength reduction method on COMSOL Multiphysics. The influence of rainfall infiltration on the stability of high rock slope in open-pit mine is analyzed.

## A new nonlinear strength reduction method

### Hoek–Brown yield criterion

Hoek–Brown (HB) strength criterion is an empirical formula of rock failure based on the theoretical research results of rock mechanics by evert Hoek and E.T. Brown. They have analyzed a large number of engineering field data. The theory scientifically sums up a large number of in-situ test results, solves many practical engineering problems, and is widely used in open-pit mining and slope engineering.

Hoek et al.^14^ introduced the geological strength index *GSI* and disturbance coefficient *D* of the rock mass into the theory. The modified generalized HB expression is as follows:

1 $$\sigma_{1} { = }\sigma_{3} { + }\sigma_{ci} \left( {m_{b} \frac{{\sigma_{3} }}{{\sigma_{ci} }} + s} \right)^{\alpha }$$

where *σ*_1_ and *σ*_3_ are the maximum and minimum principal stresses when the rock mass yields; *σ*_*ci*_ is the uniaxial compressive strength of the complete rock; *m*_*b*_, *s* and *α* are the characteristic parameters of the rock mass.

*m*_*b*_, *s* and *α* can be given as functions related to *GSI* and disturbance coefficient *D* of rock mass:

2 $$m_{b} = m_{i} \exp \left( {\frac{GSI - 100}{{28 - 14D}}} \right)$$

3 $$s = \exp \left( {\frac{GSI - 100}{{9 - 3D}}} \right)$$

4 $$\alpha = \frac{1}{2} + \frac{1}{6}\left( {e^{ - GSI/15} - e^{ - 20/3} } \right)$$

where *m*_*i*_ is Hoek Brown constant of complete rock; *GSI* is geological strength index of rock mass, which is selected according to rock mass structure and surface characteristics of structural plane; *D* is disturbance coefficient, which is 1.0 for conventional blasting, 0.7 for mechanical excavation and 0.8–0.9 for controlled blasting.

### Equivalent Mohr–Coulomb strength parameters

**Figure 1:**
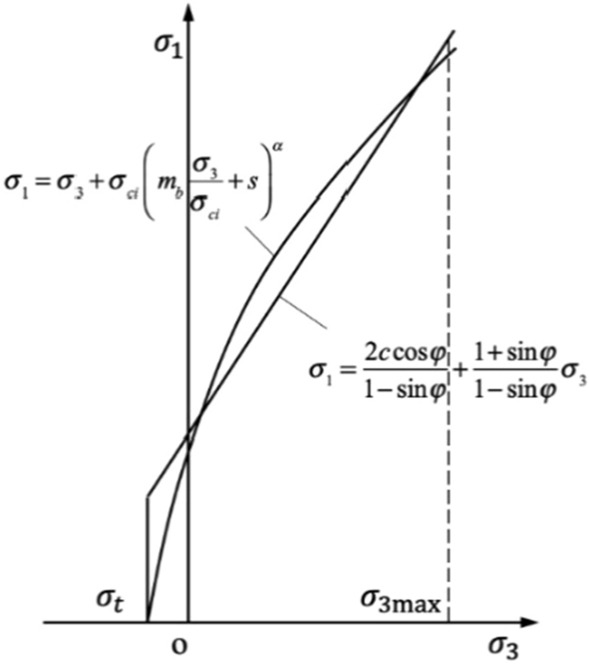
Relationships between HB and equivalent MC criteria.

Because most of the geotechnical engineering software is still based on Mohor-Coulomb (MC) failure criterion, few has been studied on non-linear shear strength reduction techniques^35–40^. It is necessary to determine the cohesion and internal friction angle of rock mass by HB criterion. HB criterion is non-linear, while MC criterion is linear, so cohesion and internal friction angle cannot be calculated directly^41–45^. Therefore, the strength curve of HB criterion is fitted as equivalent MC straight line to obtain cohesion and internal friction angle of rock mass, as shown in Fig. 1.

In the fitting process, the upper and lower difference areas of MC straight line are balanced to obtain the equivalent MC parameters.

5 $$\varphi { = }\sin^{ - 1} \left[ {\frac{{6am_{b} \left( {s + m_{b} \sigma_{3n} } \right)^{\alpha - 1} }}{{2\left( {1 + \alpha } \right)\left( {2 + \alpha } \right) + 6am_{b} \left( {s + m_{b} \sigma_{3n} } \right)^{\alpha - 1} }}} \right]$$

6 $$c = \frac{{\sigma_{c} \left[ {\left( {1 + 2\alpha } \right)s + (1 - \alpha )m_{b} \sigma_{3n} } \right]\left( {s + m_{b} \sigma_{3n} } \right)^{\alpha - 1} }}{{(1 + \alpha )(2 + \alpha )\sqrt {1 + \left[ {6\alpha m_{b} \left( {s + m_{b} \sigma_{3n} } \right)^{\alpha - 1} } \right]/\left[ {(1 + \alpha )(2 + \alpha )} \right]} }}$$

7 $$\sigma_{cm} = \sigma_{ci} \frac{{\left[ {m_{b} + 4s - \alpha \left( {m_{b} - 8s} \right)} \right]\left( {m_{b} /4 + s} \right)^{\alpha - 1} }}{{2\left( {1 + \alpha } \right)\left( {2 + \alpha } \right)}}$$

According to the actual situation of slope engineering, Hoke et al. made specific provisions for the parameters in Formula (5)–(7):

8 $$\frac{{\sigma_{3\max } }}{{\sigma_{cm} }} = 0.72\left( {\frac{{1000\sigma_{cm} }}{\gamma H}} \right)^{ - 0.91}$$

9 $$\sigma_{3n} = \frac{{\sigma_{3\max } }}{{\sigma_{ci} }}$$

where *σ*_cm_ is the compressive strength of rock mass, *σ*_3max_ is the maximum confining pressure, *H* is the height of slope and *γ* is the weight of rock mass.

**Figure 2:**
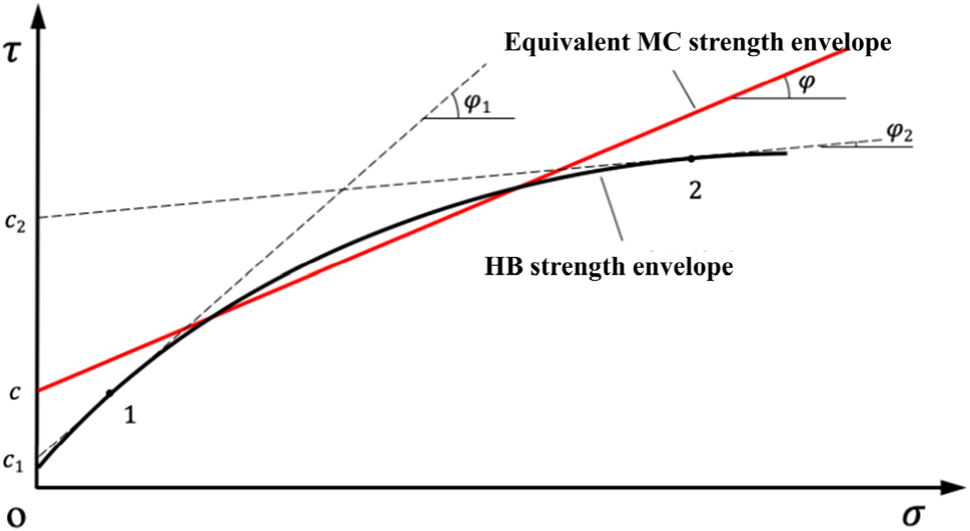
The relationship between the equivalent MC strength envelope curve and HB strength envelope curve.

As shown in Fig. 2, in the coordinate space with normal stress *σ* as the horizontal axis and shear stress *τ* as the vertical axis, the equivalent MC strength envelope curve is a straight line and HB strength envelope is a kinked line. The instantaneous cohesion *c*_*i*_ and internal friction angle *φ*_*i*_ can be obtained by making a tangent line on the HB strength envelope curve^33,35^. It can be seen from the Fig. 2 that with the increase of stress, the cohesion *c*_*i*_ increases gradually, then it will be equal to the equivalent cohesion at a certain stress, while the internal friction angle *φ*_*i*_ decreases gradually, then it will also be equal to the internal friction.

In slope engineering, the stress is relatively small in the shallow part of the slope as shown in point 1 in Fig. 2, the cohesion and internal friction angle can be *C*_1_ and *φ*_1_ by tangent line, *C*_1_ is smaller than *C*, and *φ*_1_ is larger than *φ*. However, in the deep part of the slope, the stress is relatively large as shown in point 2 in Fig. 2, the cohesion and internal friction angle can be obtained by tangent line, which are *C*_2_ and *φ*_2_ respectively, *C*_2_ is larger than *C*, and *φ*_2_ is less than *φ*.

Therefore, in view of the high stress characteristics of high rock slope, the establishment of the relationship between cohesion, internal friction angle and minimum principal stress is the key of slope strength reduction calculation.

### Nonlinear strength reduction method

The equivalent MC strength parameters are fitted in the range of *σ*_*t*_ < *σ*_3_ < *σ*_3max_. When the minimum principal stress is small, the calculated strength parameters are relatively accurate, but the larger the minimum principal stress is, the lower the calculation accuracy is. Because of the high and steep rock slope in open pit, it is necessary to determine the strength parameters of rock mass in case of large stress.

**Figure 3:**
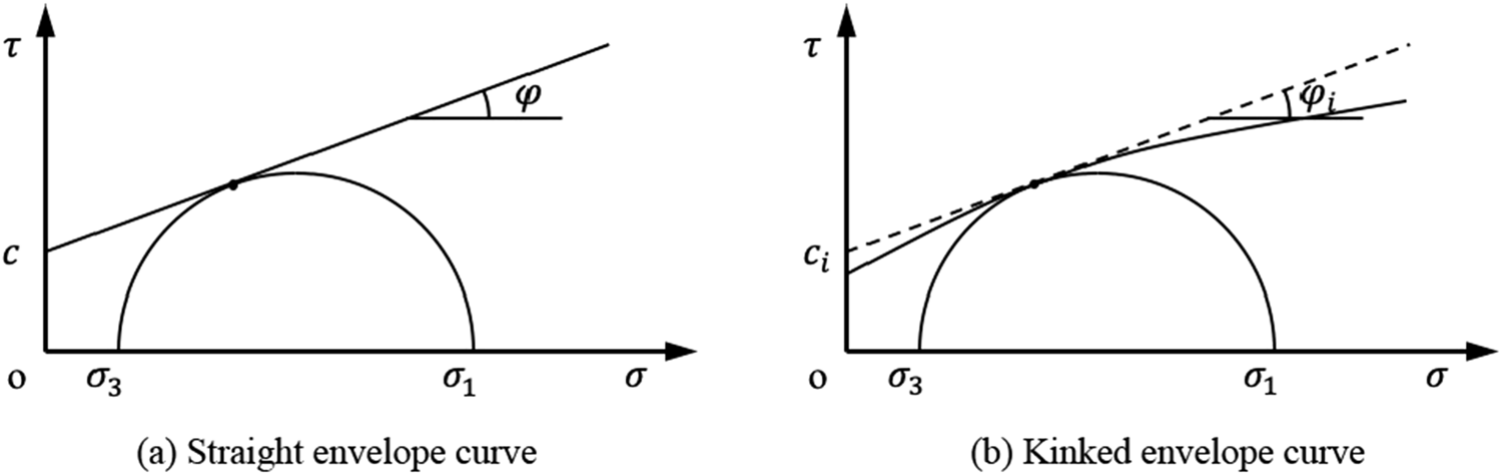
Relationships between the Mohr circle and the damage envelope.

Under the condition of complex stress, the stress state of yield point in rock mass can be represented by limit Mohr circle on the coordinate space with normal stress *σ* as horizontal axis and shear stress *τ* as vertical axis. Whether the strength envelope is a straight or kinked line, only one point of the limit Mohr circle is tangent to the envelope curve, as shown in Fig. 3.

The cohesion and internal friction angle corresponding to any point on the HB strength envelope curve are expressed by *c*_*i*_ and *φ*_*i*_ respectively. The normal stress *σ* and shear stress *τ* on this point can be expressed by MC theory as follows:

10 $$\sigma { = }\frac{{\sigma_{1} { + }\sigma_{3} }}{2} - \frac{{\sigma_{1} - \sigma_{3} }}{2}\sin \varphi_{i}$$

11 $$\tau { = }\frac{{\sigma_{1} - \sigma_{3} }}{2}\cos \varphi_{i}$$

The generalized HB expression Formula (1) is substituted into Formula (10) and Formula (11) yields:

12 $$\sigma { = }\frac{{1 - \sin \varphi_{i} }}{2}\sigma_{ci} \left( {m_{b} \frac{{\sigma_{3} }}{{\sigma_{ci} }} + s} \right)^{\alpha } + \sigma_{3}$$

13 $$\tau { = }\frac{{\cos \varphi_{i} }}{2}\sigma_{ci} \left( {m_{b} \frac{{\sigma_{3} }}{{\sigma_{ci} }} + s} \right)^{\alpha }$$

For HB failure criterion, because the failure envelope curve is a kinked line, *φ*_*i*_ changes with the change of minimum principal stress state, that is, *φ*_*i*_ = *φ*(*σ*_3_).

Taking the derivative of Formula (12) and Formula (13) with respect to *σ*_3_ yields:

14 $$\frac{{{\text{d}}\sigma }}{{{\text{d}}\sigma_{3} }}{ = } - \frac{{\cos \varphi_{i} }}{2}\frac{{{\text{d}}\varphi_{i} }}{{{\text{d}}\sigma_{3} }}\sigma_{ci} \left( {m_{b} \frac{{\sigma_{3} }}{{\sigma_{ci} }} + s} \right)^{\alpha } + \frac{{1 - \sin \varphi_{i} }}{2}\alpha m_{b} \left( {m_{b} \frac{{\sigma_{3} }}{{\sigma_{ci} }} + s} \right)^{\alpha - 1} + 1$$

15 $$\frac{{{\text{d}}\tau }}{{{\text{d}}\sigma_{3} }}{ = } - \frac{{\sin \varphi_{i} }}{2}\frac{{{\text{d}}\varphi_{i} }}{{{\text{d}}\sigma_{3} }}\sigma_{ci} \left( {m_{b} \frac{{\sigma_{3} }}{{\sigma_{ci} }} + s} \right)^{\alpha } + \frac{{\cos \varphi_{i} }}{2}\alpha m_{b} \left( {m_{b} \frac{{\sigma_{3} }}{{\sigma_{ci} }} + s} \right)^{\alpha - 1}$$

On the *τ*-*σ* coordinate space:

16 $${\text{tan}}\varphi_{i} { = }\frac{{{\text{d}}\tau }}{{{\text{d}}\sigma }}{ = }\frac{{{\text{d}}\tau }}{{{\text{d}}\sigma_{3} }}/\frac{{{\text{d}}\sigma }}{{{\text{d}}\sigma_{3} }}$$

Substituting Formula (14) and Formula (15) into Formula (16) and simplifying yields:

17a $$\varphi_{i} {\text{ = sin}}^{ - 1} \left( {\frac{{\alpha m_{b} A^{\alpha - 1} }}{{2 + \alpha m_{b} A^{\alpha - 1} }}} \right)$$

17b $$A = m_{b} \frac{{\sigma_{3} }}{{\sigma_{ci} }} + s$$

The equation of MC failure envelope curve on τ—σ coordinate space can be expressed as follows:

18 $$\tau { = }c_{i} + \sigma \tan \varphi_{i}$$

Substituting Formula (12), (13) and (17) into Formula (18) yields:

19 $$c_{i} = \frac{{2\sigma_{ci} A^{\alpha } + \alpha m_{b} \sigma_{ci} A^{2\alpha - 1} }}{{2\left( {2 + \alpha m_{b} A^{\alpha - 1} } \right)\sqrt {1 + \alpha m_{b} A^{\alpha - 1} } }} - \frac{{\alpha m_{b} A^{\alpha - 1} }}{{2\sqrt {1 + \alpha m_{b} A^{\alpha - 1} } }}\sigma_{3}$$

**Figure 4:**
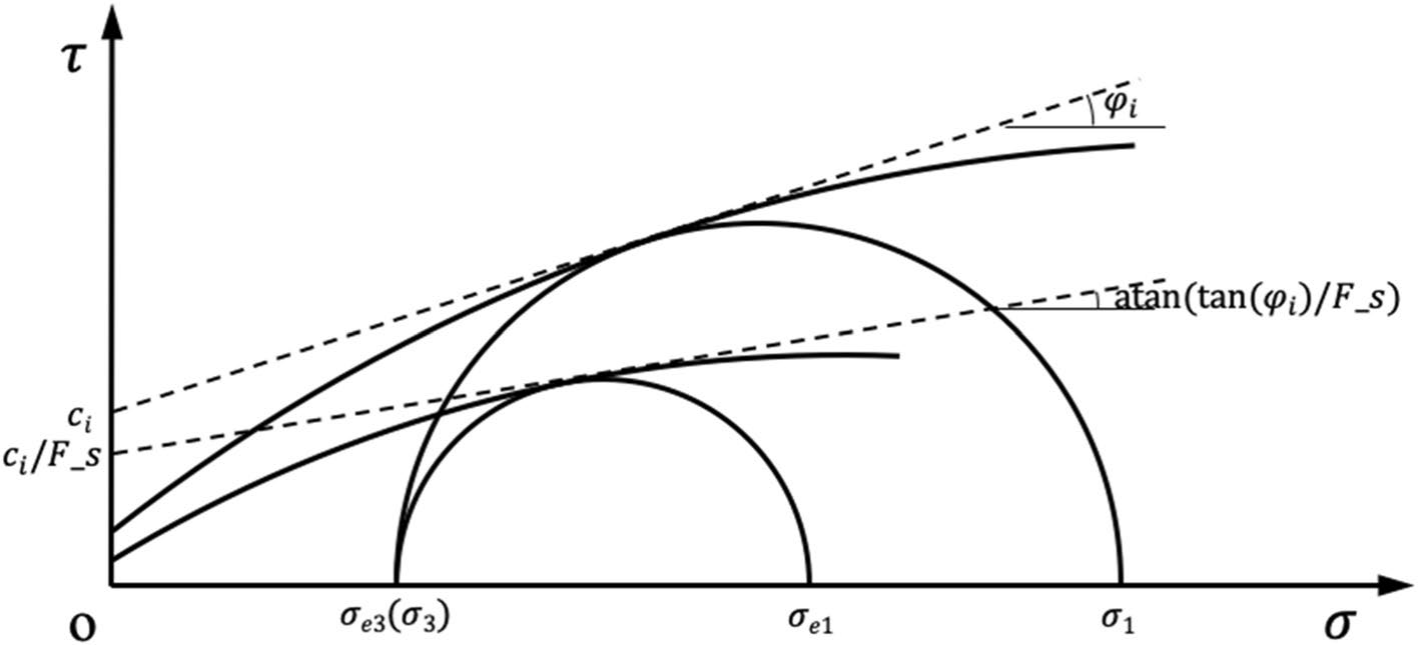
Reduction calculation sketch.

It can be seen from Formula (17) and (19) that the values of *c*_*i*_ and *φ*_*i*_ are related to the minimum principal stress in the limit state, the HB characteristic parameters of rock mass and the uniaxial compressive strength of rock. The HB characteristic parameters and uniaxial compressive strength of HB rock mass are known. The stress state of the slope rock mass is calculated by the finite element method, and the minimum principal stress *σ*_e3_ is obtained. As shown in Fig. 4, *c*_*i*_ and *φ*_*i*_ of each point in the rock mass can be obtained by substituting *σ*_3_ = *σ*_e3_.

### Implementation of nonlinear strength reduction method based on HB criterion

**Figure 5:**
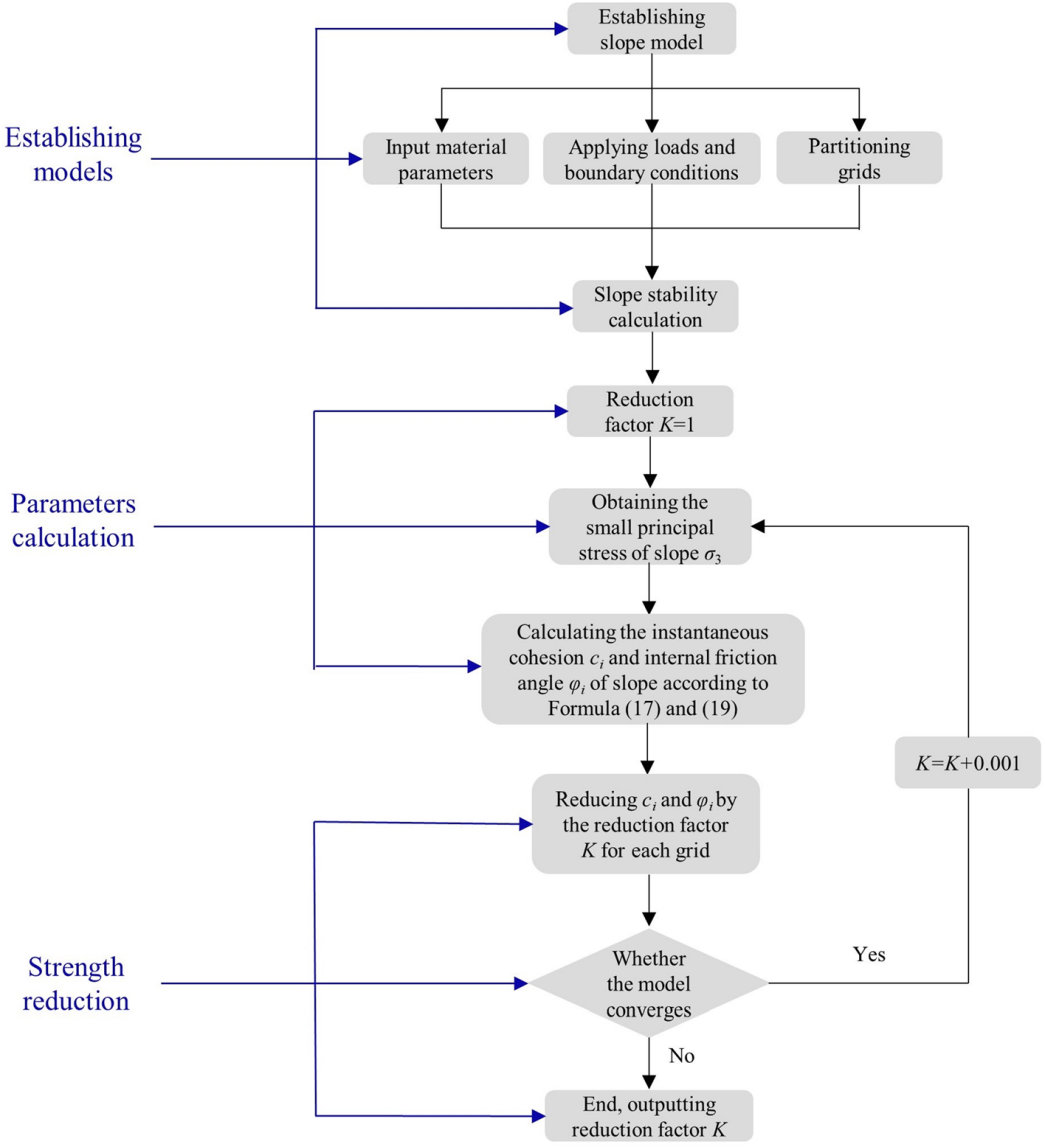
Flow chart for calculation of strength reduction method.

Using COMSOL multiphysics software, this paper embeds the calculation method of instantaneous equivalent MC strength parameters into the strength reduction calculation program, so as to realize the application of HB criterion finite element strength reduction method in slope stability analysis. The calculation flow is shown in Fig. 5. The calculation process consists of three parts: establishing models, parameters calculation and strength reduction. In the calculations, the instantaneous internal friction angle and instantaneous cohesion are derived according to Hoek–Brown criterion, while the elastic–plastic finite element calculation of slope and minimum principal stress are calculated according to Mohr–Coulomb criterion. The proposed method includes the following steps:

- *Step 1* The solution area is determined according to the geometric parameters of slope, and the material parameters of rock mass are inputted. Mesh generation is carried out according to certain laws in the solution area, and the triangular element with three nodes is selected. The loading and boundary conditions are set, and the initial slope stability can be calculated.
- *Step 2* The initial value of reduction coefficient K is set to 1, the stress state of slope rock mass is calculated by FEM. The minimum principal stresses for each element are obtained as the initial conditions for the elasto-plastic calculations. Substituting the known Hoek–Brown parameters and the minimum principal stress into Formulas (17) and (19), the instantaneous cohesion ci and internal friction angle φi of each element of the slope model are calculated.
- *Step 3* According to the reduction coefficient K, ci and φi are reduced. Judge whether the slope model converges. If the model converges, end the calculation and output the reduction coefficient K. Otherwise, according to the reducted parameters, the Mohr–Coulomb criterion is used for the elastic–plastic finite element calculation of slope to obtain the minimum principal stress of each element, and then instantaneous strength parameters are continuously adjusted in Step 2. The calculation procedure in Steps 2–3 is repeated until slope failure occurs.

## Case study and verification

**Figure 6:**
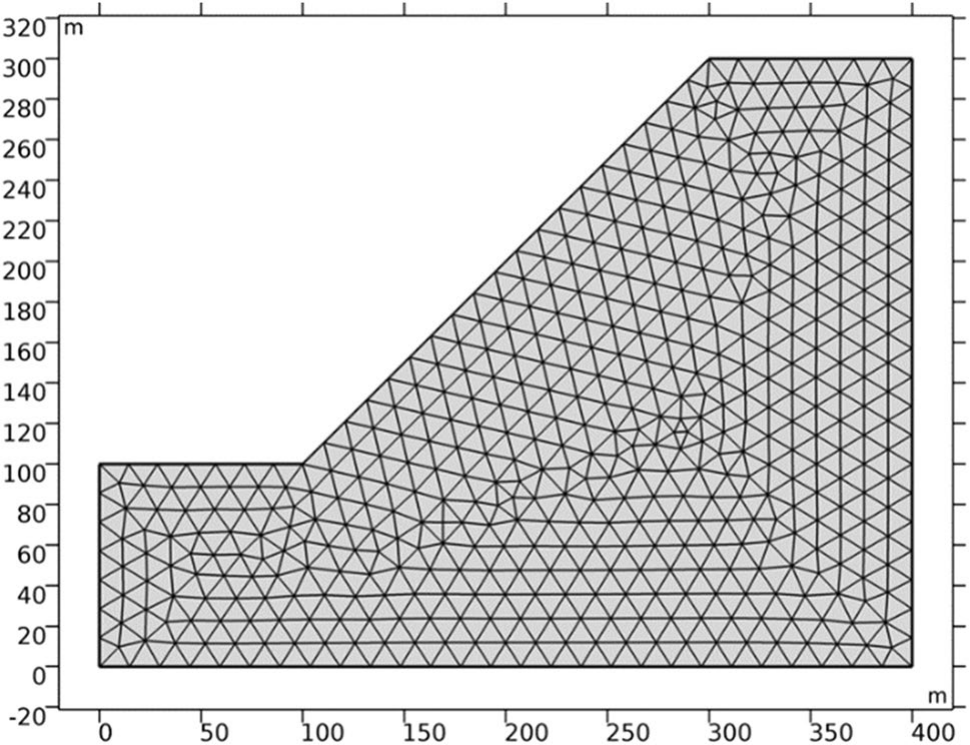
Slope model establishment and grid division.

In order to verify the effectiveness of the new nonlinear strength reduction method proposed in the previous section, a simple slope model is established using COMSOL multiphysics software, with a slope height of 200 m and a slope gradient of 1:1, as shown in Fig. 6. The model boundary conditions are set as followed: the left and right boundaries of the slope are set as roller support constraints, and the bottom boundary is set as fixed constraints. Case A adopts the new nonlinear strength reduction method proposed in the previous section, and case B adopts the equivalent MC strength parameter reduction.

**Figure 7:**
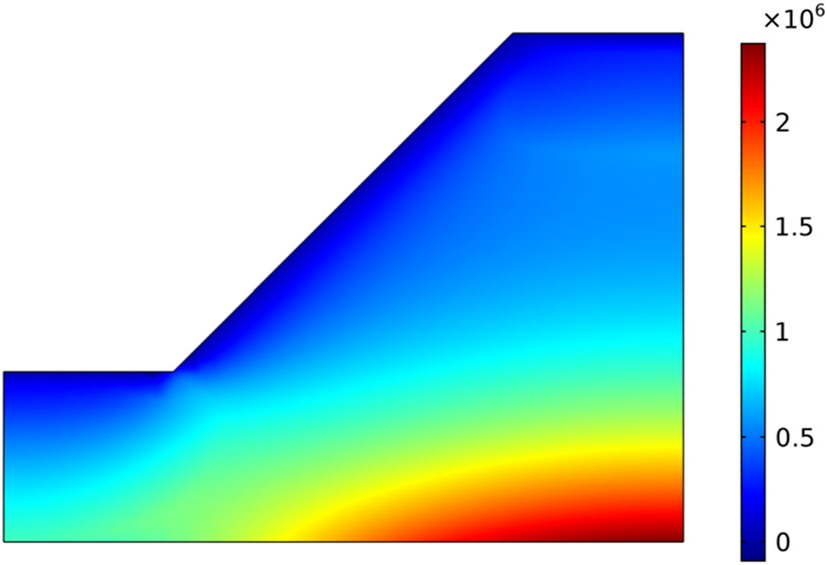
Minimum principal stress (N/m^2^).

The strength parameters of slope rock mass are based on the data in Table 2, and the strength parameters of equivalent MC criterion are calculated according to Formula (5) and Formula (6). The stress state in the slope is calculated using COMSOL multiphysics software, and the calculation result of the minimum principal stress *σ*_3_ is shown in Fig. 7.

**Table 2:** Strength parameter.

HB strength parameters							Equivalent MC strength parameters	
*σ*_*ci*_ /MPa	*GSI*	*D*	*m*_*i*_	*m*_*b*_	*S*	*α*	*c* /KPa	*φ*
70	30	0.8	10	0.155	2.476E-5	0.522	456.7	23.14°

**Figure 8:**
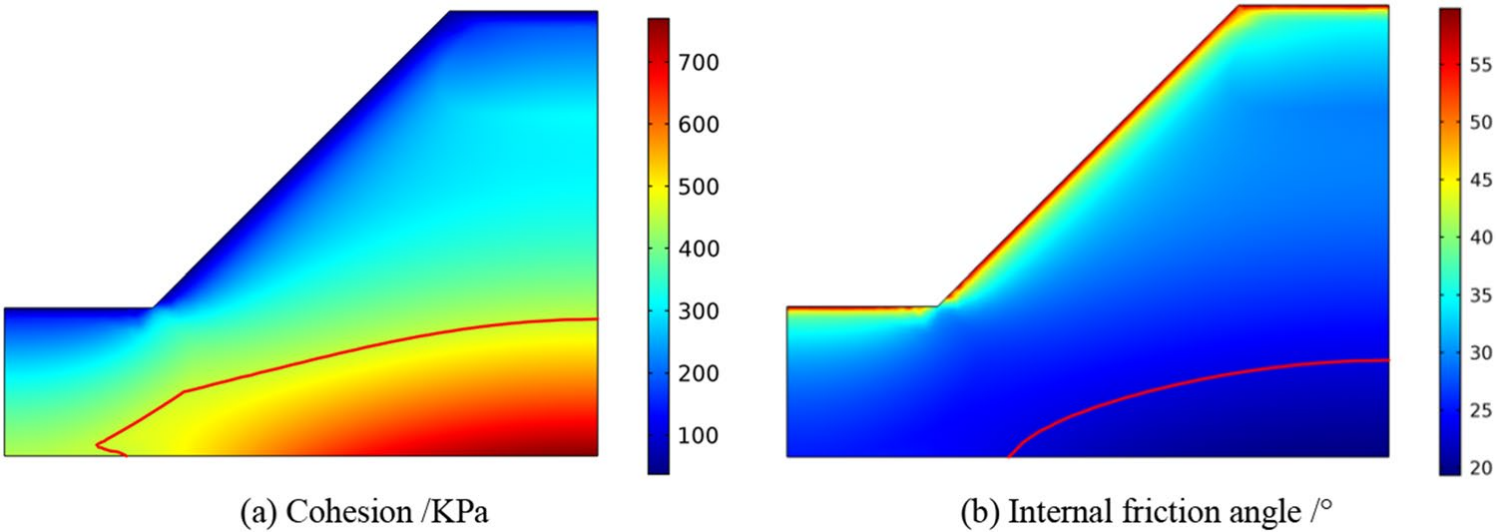
The distribution of cohesion and internal friction angle.

The calculated minimum principal stress *σ*_3_ and HB strength parameters are substituted into Formula (17) and Formula (19) to obtain the non-linear MC strength parameters *c*_*i*_ and *φ*_*i*_ in the slope. The calculation results are shown in Fig. 8.

From the calculation results shown in Fig. 8, it can be seen that the cohesion of case A decreases gradually from the inside out, and the cohesion at the upper part is less than the equivalent cohesion, while the change of internal friction angle is opposite to the cohesion, they are non-linear.

**Figure 9:**
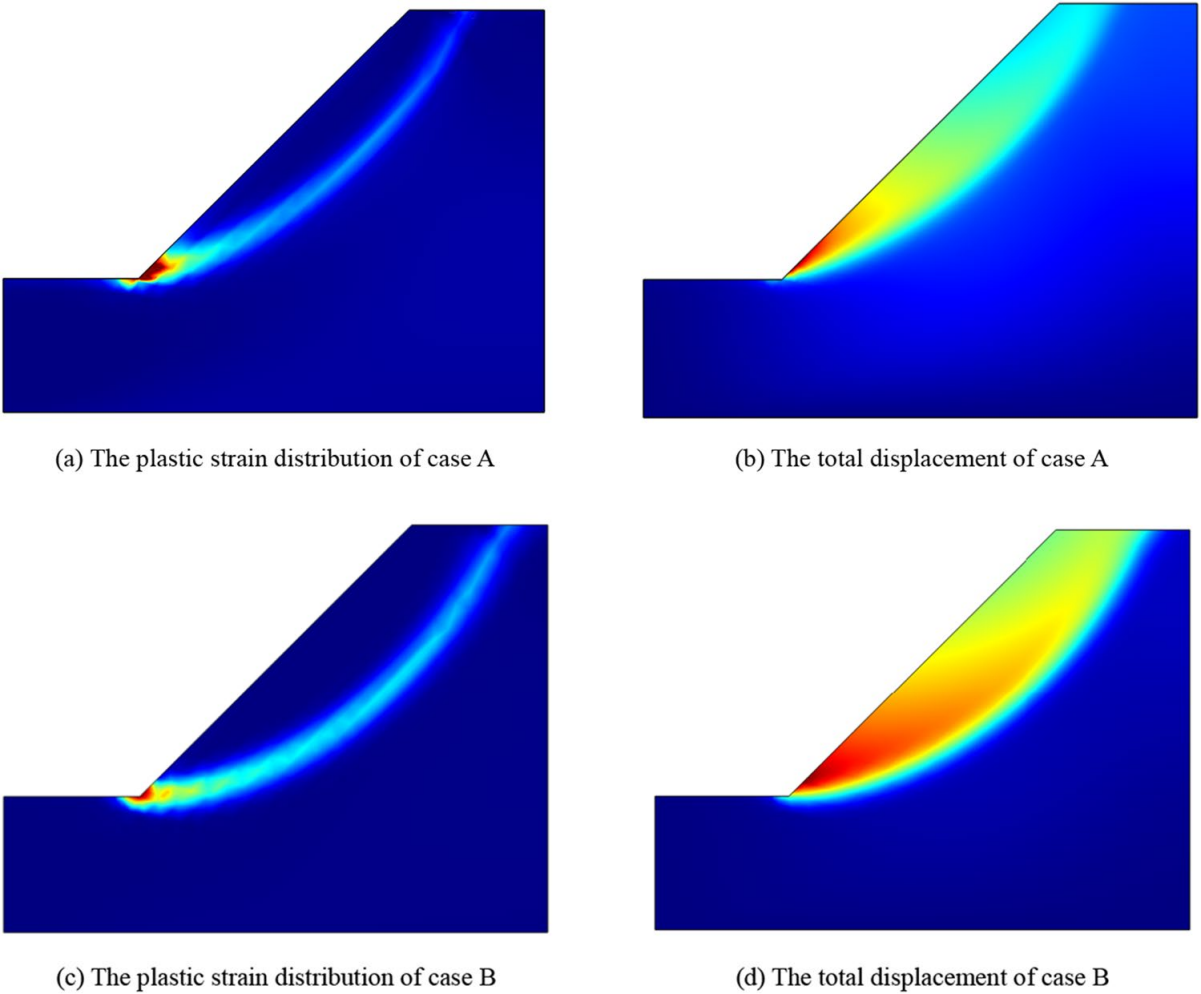
The plastic strain distribution and total displacement.

For the case A, the strength reduction calculation is carried out by using the nonlinear MC strength parameters *c*_*i*_ and *φ*_*i*_ based on HB criterion. The safety factor of the slope is 1.24. For the case B, the equivalent MC criterion strength parameters are directly used for strength reduction calculation. The safety factor of the slope is 1.30. The plastic strain distribution and total displacement diagram of the two cases in the limit state are shown in Fig. 9.

It can be clearly observed from Fig. 9 that the potential sliding surface of case A is closer to the slope surface than case B, and the landslide body of case A is smaller than case B. And the factor of safety (FOS) calculated in case A is less than that calculated in case B and the stability of slope in case B is overestimated. Obviously, the simulation results of case A are more closer to the actual situation. Therefore, the proposed nonlinear strength reduction method is more reasonable in numerical calculation.

The equivalent MC strength parameters are estimated by fitting, and their accuracy depends on the range of the minimum principal stress. However, the nonlinear strength parameters derived in the previous section are related to the minimum principal stress, the characteristic parameters of HB rock mass and the uniaxial compressive strength of rock. For the strength reduction of high rock slope in open pit mines, the stress state should be considered to propose the reduction method. The results show that it is more reasonable to simulate and calculate high rock slope with the proposed nonlinear strength reduction method, which can be effectively applied to the stability analysis of high rock slope.

**Figure 10:**
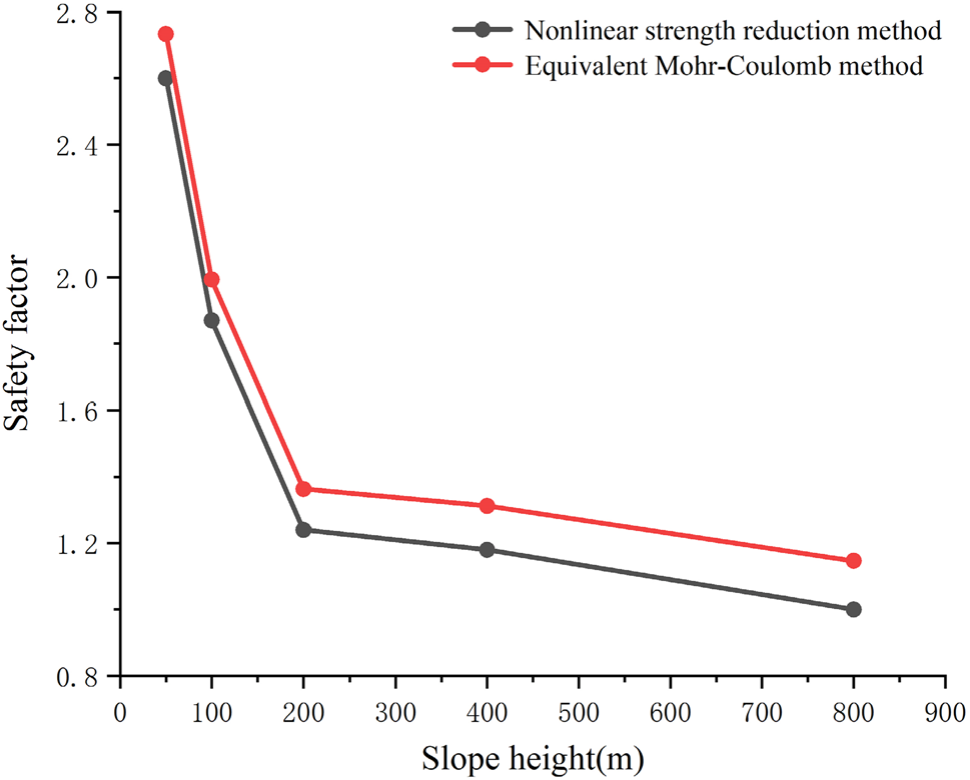
Relation curve between slope height and safety factor.

In order to further explore the rationality and practicability of the proposed nonlinear strength reduction method for the stability calculation of high rock slope, the authors established rock slope with different height in the COMSOL multiphysics software, and analyzed the difference of the factor of safety (FOS) calculated using the two methods with the change of slope height, as shown in Fig. 10. Figure 10 shows the relationship between safety factor and slope height. It can be seen from the figure that as the height increases, the safety factor of the whole slope decreases gradually. In the meantime, when the slope is low, the difference between the two safety factors is small, but with the increase of the slope height, the difference between the calculation results of the equivalent Mohr- Coulomb method and the nonlinear strength reduction method gradually increases. The characteristics of high rock slope with a wide range of stress variation is not fully considered in the equivalent Mohr- Coulomb method and the slope stability calculation results may be overestimated. Therefore, the nonlinear strength reduction method based on HB criterion could be effectively applied to the stability analysis of high rock slope.

## Application on southwest slope of Dagushan Open-pit Iron Mine

### The effect mechanism of rainfall infiltration

**Figure 11:**
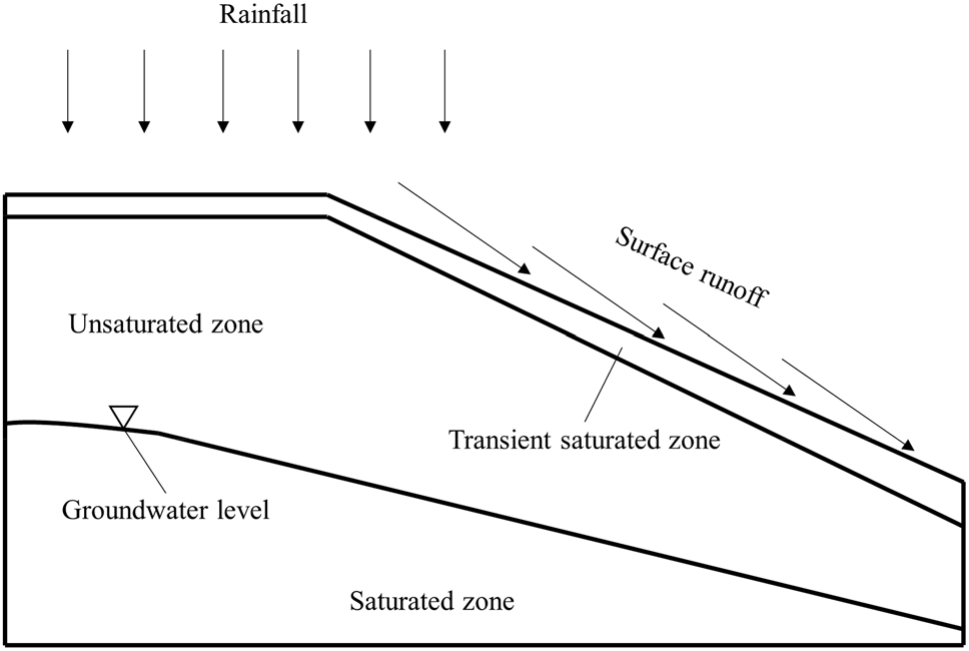
Rainfall infiltration of slope.

Rainfall infiltration is the process of rainwater infiltration through the surface into rock mass, which is essentially the movement process of rainwater in the porous medium constantly displacing air. The saturated zone is below the water table, and the shallow surface layer will evolve into a transient saturated zone under the influence of rainfall. The unsaturated zone is between transient saturated zone and water table, as shown in Fig. 11. Overall, the effect mechanism of rainfall infiltration on the stability of rock slope includes two aspects. On the one hand, rainfall can cause the groundwater level to rise, leading to a transient saturated zone in the surface layer of slope and raising the pore-water pressure in the corresponding area. On the other hand, rock mass can be affected by the softening effect of rainwater. Rainfall will lead to a decrease in the rock strength and reduce the effective stress inside rock, thus inducing geological disasters such as landslides and mudslides.

### Geological conditions of Dagushan Iron Mine

Dagushan Iron Mine is located in Anshan City, Liaoning Province, China. The mine is located in the edge of Qianshan Mountain and the branch of Changbai Mountain. Its landform is mainly hilly. Because the southeast is Qianshan Mountain area and the northwest is mainly plain, the terrain is low in the northwest and high in the southeast. According to the regional geological data and survey report, the strata from top to bottom in the mining area are mainly: Quaternary eluvium of Cenozoic, quartz layer of Diaoyutai formation of Qingbaikou system of Upper Proterozoic, qianmei layer of Langzishan formation of Liaohe Group of Lower Proterozoic, chlorite quartz sheet layer and iron quartz layer of yingtaoyuan formation of Anshan Group of Archaean. The pit of Dagushan Iron Mine is located in the west of the mining area. The mining technology is high and steep slope mining, and the open pit mining extends to the underground in a ladder shape. The southwest side of Dagushan iron mine pit is a typical high rock slope. Previous landslides were caused by sliding along the weak structural plane caused by precipitation. The annual rainy season is the most frequent period of landslide disasters.

### The establishment of geometric model

**Figure 12:**
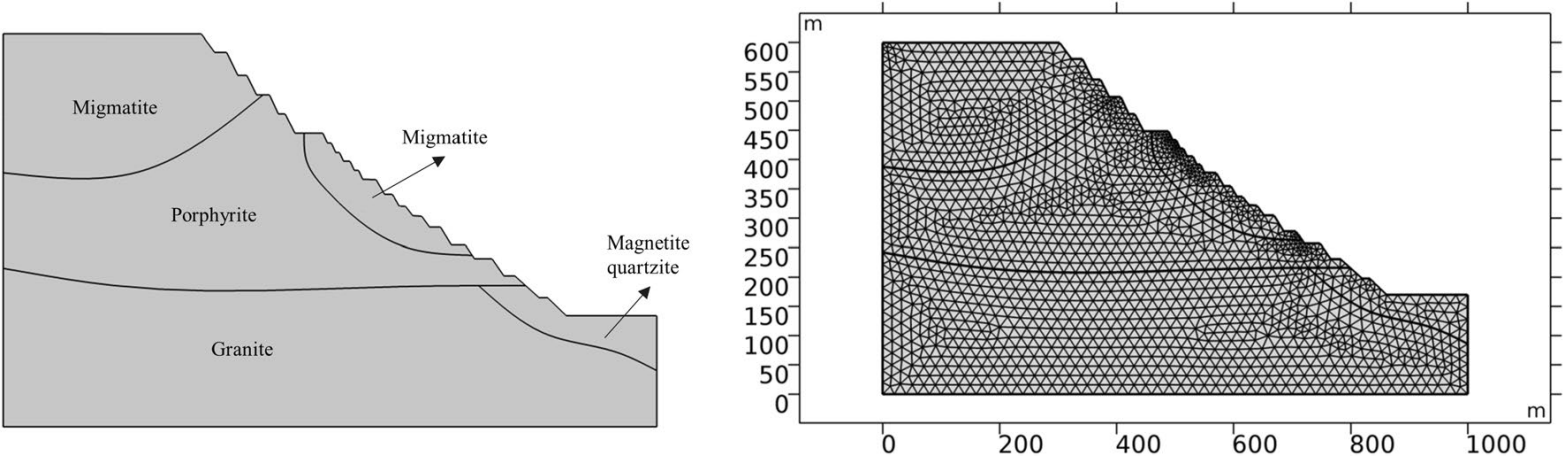
Strata and Calculation model of the slope.

Based on the field investigation of Dagushan Open-pit Iron Mine and the collection of relevant geological data and monitoring data, the southwest slope area is selected as the research object, which is a typical high rock slope. The main rock layers of the slope in this area are migmatite, porphyrite, granite and magnetite quartzite. The distribution of the rock layers in the section is shown in Fig. 12. The stratum in this area is composed of many kinds of rock strata, and the joint fissures are relatively developed. Through communication with the site staff, there has been a small-scale slide on the slope in the area, which causes the deformation of the upper part of the slope and damages the corresponding facilities and buildings. According to the microseismic monitoring data, a large number of microseismic events occurred in this area due to rainfall.

In this work, COMSOL multiphysics multi physical field simulation software is used for numerical calculation, and Richards equation model is used for seepage field calculation. The selected calculation profile of the southwest slope of Dagushan open-pit iron mine is shown in Fig. 12. The whole calculation grid area is 411,500 square meters, the unit type is triangle unit, the number of units is 3245, and the number of grid vertices is 1719.

### Initial conditions and parameters

#### Permeability tensor and hydraulic characteristic parameters of fractured rock mass

Through the field sampling and investigation of the joints and fissures in the slope rock mass of Dagushan open-pit mine, and then statistical analysis, the fracture geometric parameters of the southwest slope rock mass are obtained as shown in Table 3.

**Table 3:** Fracture geometric parameters.

Rock type	Fracture group	Average hydraulic clearance width (mm)	Fissure distance (m)	Dip angle (º)
Migmatite	1	3.0	3.8	115
	2	2.0	6.7	48
	3	0.8	8.4	158
Porphyrite	1	4.0	5.2	121
	2	2.0	7.8	67
Granite	1	0.5	4.9	28
	2	0.9	11.2	81
Magnetite quartzite	1	0.3	6.0	50
	2	0.6	8.9	113

According to the fracture geometric parameters in Table 2, the equivalent permeability tensor of the corresponding rock mass is calculated by the fracture sampling measurement method, and the calculation results of the equivalent permeability tensor of each rock layer are shown in Table 4.

**Table 4:** Equivalent permeability tensor of rock mass.

Rock type	Equivalent permeability tensor (m/s)
Migmatite	$$\left[ {\begin{array}{*{20}c} {1.516 \times 10^{ - 6} } & {1.755 \times 10^{ - 6} } \\ {1.755 \times 10^{ - 6} } & {5.312 \times 10^{ - 6} } \\ \end{array} } \right]$$
Porphyrite	$$\left[ {\begin{array}{*{20}c} {2.794 \times 10^{ - 6} } & {4.136 \times 10^{ - 6} } \\ {4.136 \times 10^{ - 6} } & {8.095 \times 10^{ - 6} } \\ \end{array} } \right]$$
Granite	$$\left[ {\begin{array}{*{20}c} {1.754 \times 10^{ - 8} } & { - 1.685 \times 10^{ - 8} } \\ { - 1.685 \times 10^{ - 8} } & {5.645 \times 10^{ - 8} } \\ \end{array} } \right]$$
Magnetite quartzite	$$\left[ {\begin{array}{*{20}c} {4.544 \times 10^{ - 9} } & {5.319 \times 10^{ - 9} } \\ {5.319 \times 10^{ - 9} } & {1.895 \times 10^{ - 8} } \\ \end{array} } \right]$$

The water retention curves are fitted by the Van Genuchten model, which can be expressed as:

20 $$\Theta = \frac{{\theta_{w} - \theta_{r} }}{{\theta_{s} - \theta_{r} }} = \left[ {\frac{1}{{1 + \left( {\alpha \psi } \right)^{n} }}} \right]^{m}$$

where $\Theta$ is the saturation; $\theta_{w}$ is the moisture rate of rock mass; $\theta_{s}$ is the saturated moisture rate of rock mass; $\theta_{r}$ is the residual moisture rate of rock mass; $\psi$ is the matrix suction; $\alpha$, $n$ and $m$ are characteristic parameters, and $m = 1 - {1 \mathord{\left/ {\vphantom {1 n}} \right. \kern-\nulldelimiterspace} n}$.

The fitted hydraulic characteristics of rock slope are shown in Table 5.

**Table 5:** Fitted hydraulic characteristics.

Rock type	$\theta_{s}$	$\theta_{r}$	α (kPa^−1^)	*n*	*m*
Migmatite	0.17	0.03	4.2 × 10^–3^	1.9	0.4737
Porphyrite	0.14	0.05	8.5 × 10^–3^	1.7	0.4118
Granite	0.09	0.02	11.3 × 10^–3^	2.3	0.5652
Magnetite quartzite	0.07	0.01	14.0 × 10^–3^	2.0	0.5000

#### Mechanical parameters of rock mass

Through field investigation and data collection, the development of joint fissures, dominant joint occurrence and structural plane distribution characteristics of slope rock mass can be obtained. According to the rock mechanics indexes obtained from various rock mechanics tests conducted before in Dagushan, the calculation parameters of slope stability are obtained based on HB strength theory. The adopted values of relevant calculation parameters are shown in Table 6.

**Table 6:** Parameters for stability calculation.

Rock type	Density (kg/m^3^)	Uniaxial compression stress (MPa)	Elastic modulus (GPa)	Poisson's ratio	Hoek–Brown strength parameters		
					*GSI*	*D*	*m*_*i*_
Migmatite	2600	62.29	38.72	0.33	35	0.9	12
Porphyrite	2700	79.61	41.19	0.28	45	0.8	15
Granite	2700	83.55	54.13	0.20	50	0	18
Magnetite quartzite	3000	109.32	77.42	0.11	50	0.9	20

#### Rainfall schemes

The change of rainfall conditions will affect the distribution of seepage field in the rock slope, and the seepage field will affect the stability of the slope. In this work, we will study the change of slope seepage field and then calculate the change of slope stability under different rainfall conditions. According to the relevant meteorological data, the daily maximum precipitation of Dagushan open-pit mining area is 236.8 mm. On this basis, to analyze the influence of rainfall intensity, rainfall duration and total rainfall on slope stability, we designed four different schemes as shown in Table 7.

**Table 7:** Rainfall schemes.

Scheme	Scheme I	Scheme II	Scheme III	Scheme IV
Rainfall intensity (mm/h)	10	10	20	40
Rainfall duration (h)	12	24	12	6
Duration after rain stops (h)	24	24	24	24
Total rainfall (mm)	120	240	240	240

#### Initial conditions

The boundary of the left and right sides of the slope numerical model is set with a given water head boundary, the water head is equal to the height of the groundwater level at the boundary. In the meantime, the bottom boundary is set as flux boundary due to hydraulic conduction. The boundary conditions of the slope surface are set according to the rainfall intensity: when the rainfall intensity is less than the saturated permeability coefficient of the slope rock, the surface boundary is set as a given flow boundary, and the flow size is rainfall intensity; if not, the boundary is set as the given water head boundary, and the surface runoff formed at this time is relatively small, so the water head height is the elevation of this place, that is, the surface of the slope is the zero-pressure surface.

**Figure 13:**
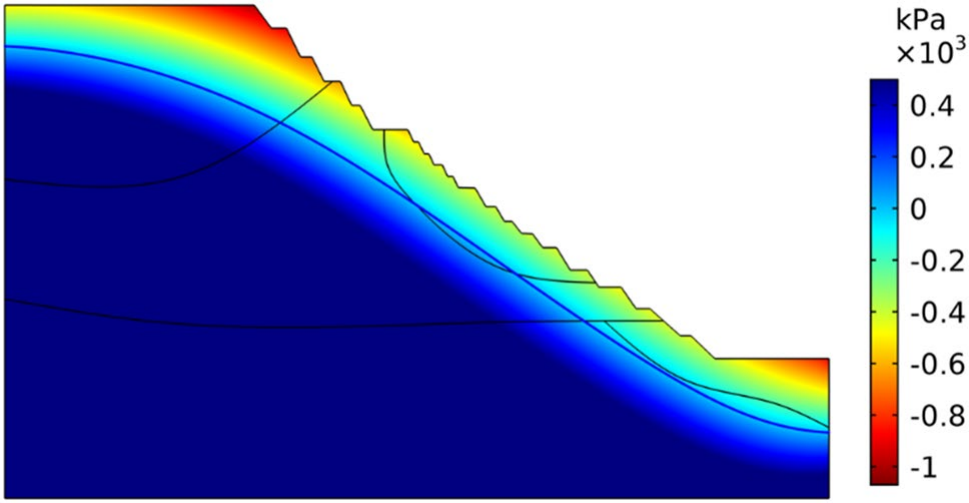
Initial pore water pressure and groundwater level.

The initial conditions are obtained from the hydrogeological conditions of the region according to the back analysis of the stable seepage field, and the pore water pressure distribution in line with the actual situation is obtained by continuously adjusting the flow flux at the bottom boundary. The calculated initial pore water pressure distribution is shown in Fig. 13, in which the blue solid line is the groundwater level line, the pore water pressure in the area below the groundwater level line is positive, and the pore water pressure in the area below the groundwater level line is negative, and the negative value represents the matrix suction in the area.

### Seepage simulation analysis of high rock slope under rainfall infiltration

**Figure 14:**
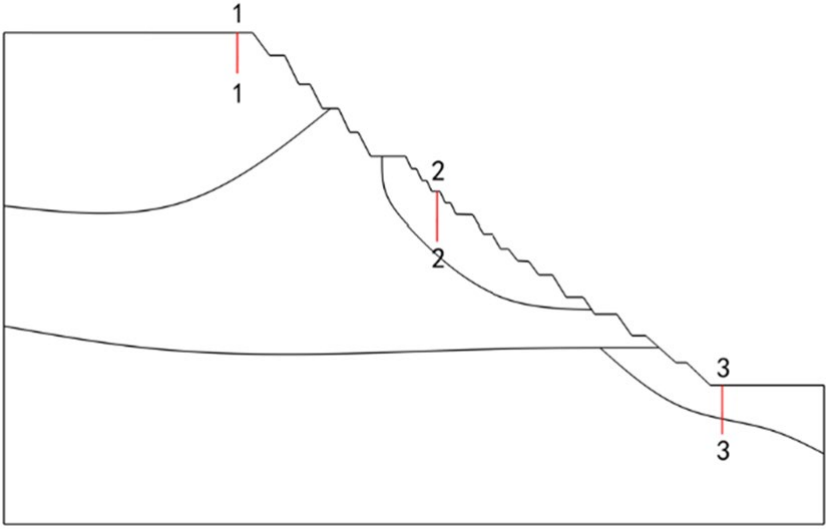
Locations of characteristic profiles.

**Figure 15:**
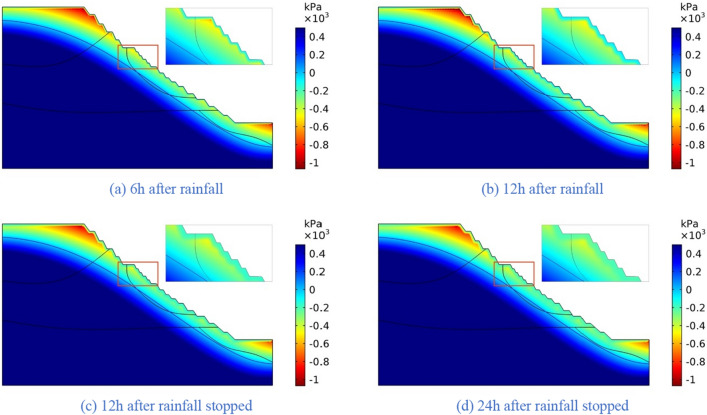
The pore-water pressure distribution (Scheme I).

**Figure 16:**
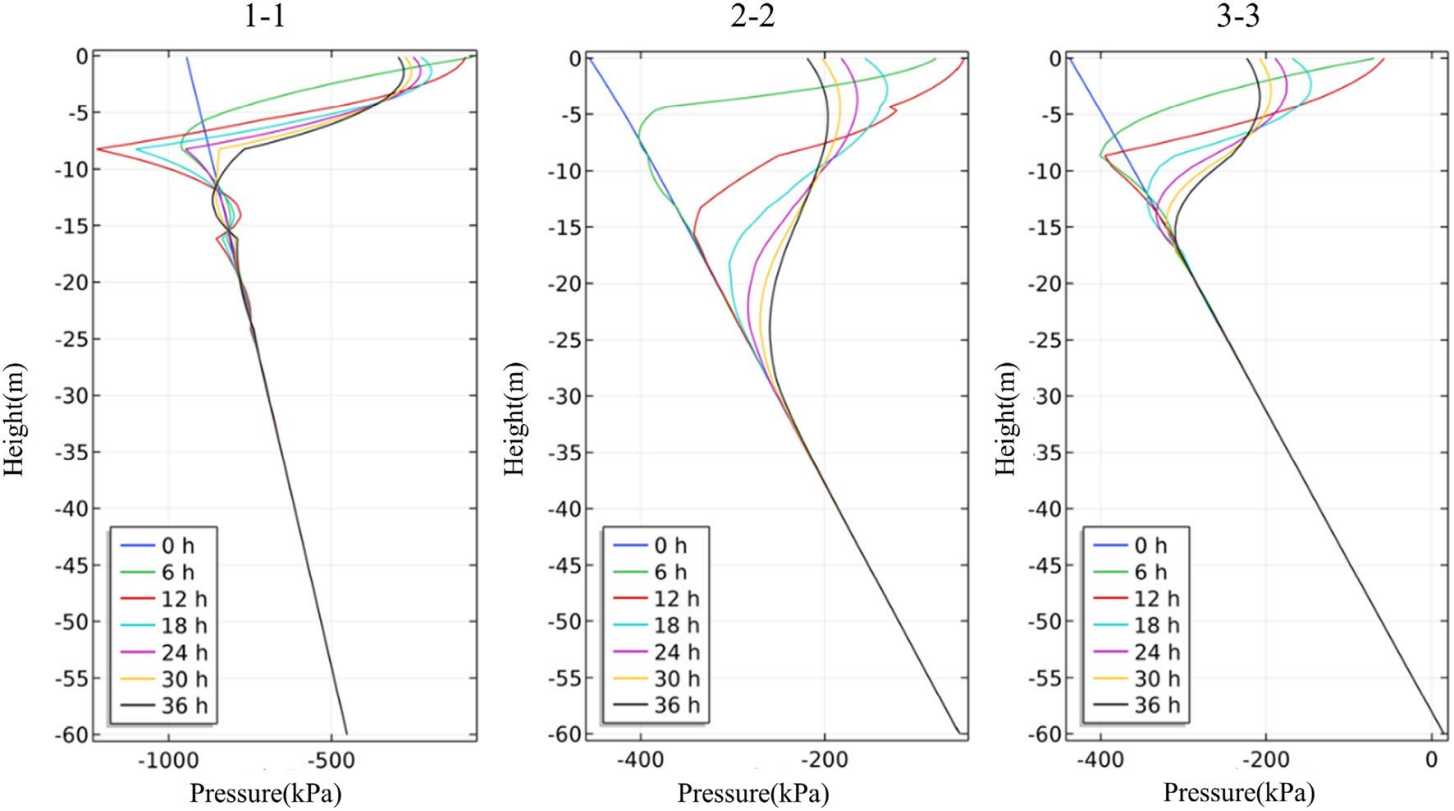
The variation of pore-water pressure in characteristic profiles(Scheme I).

**Figure 17:**
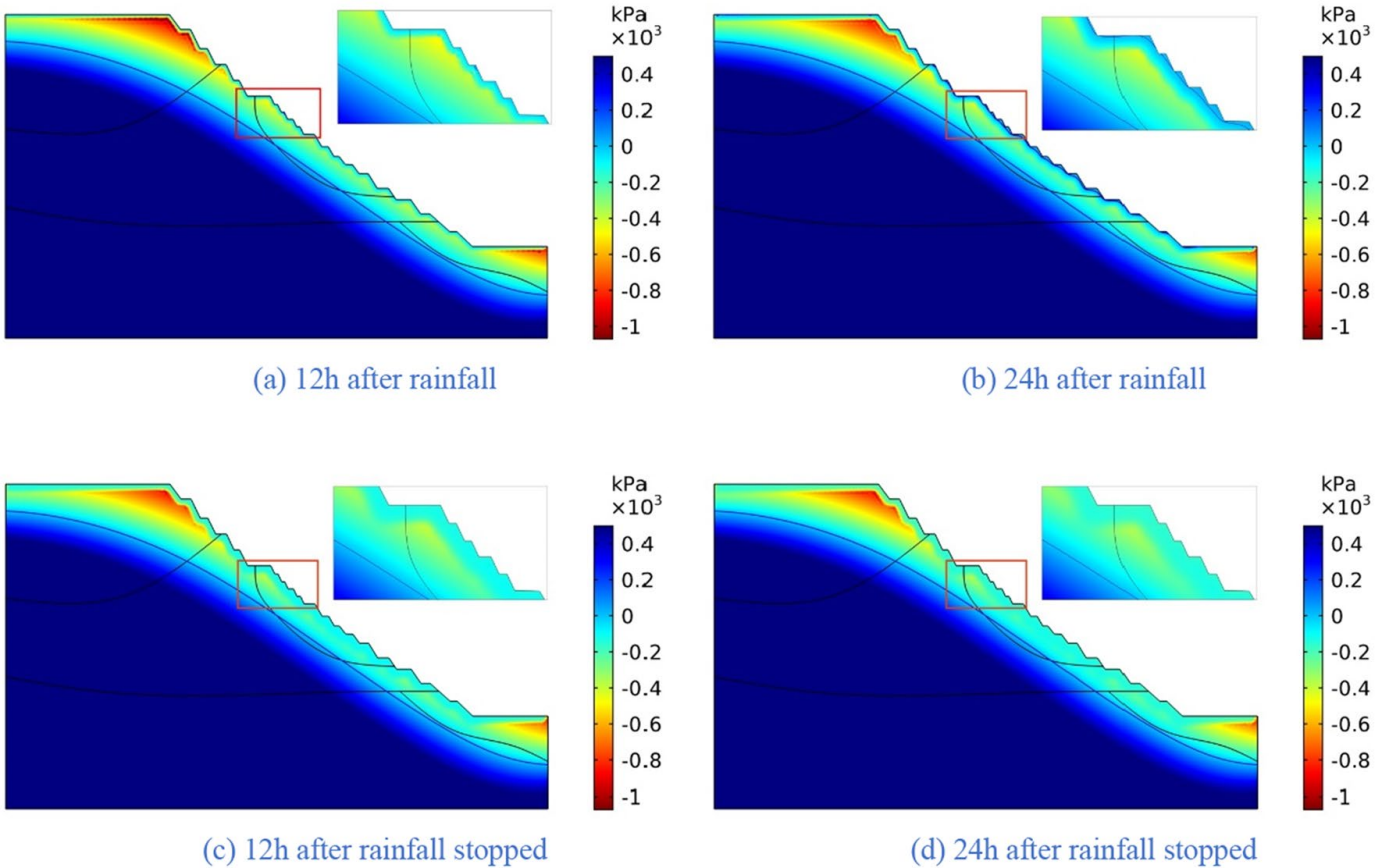
The pore-water pressure distribution (Scheme II).

**Figure 18:**
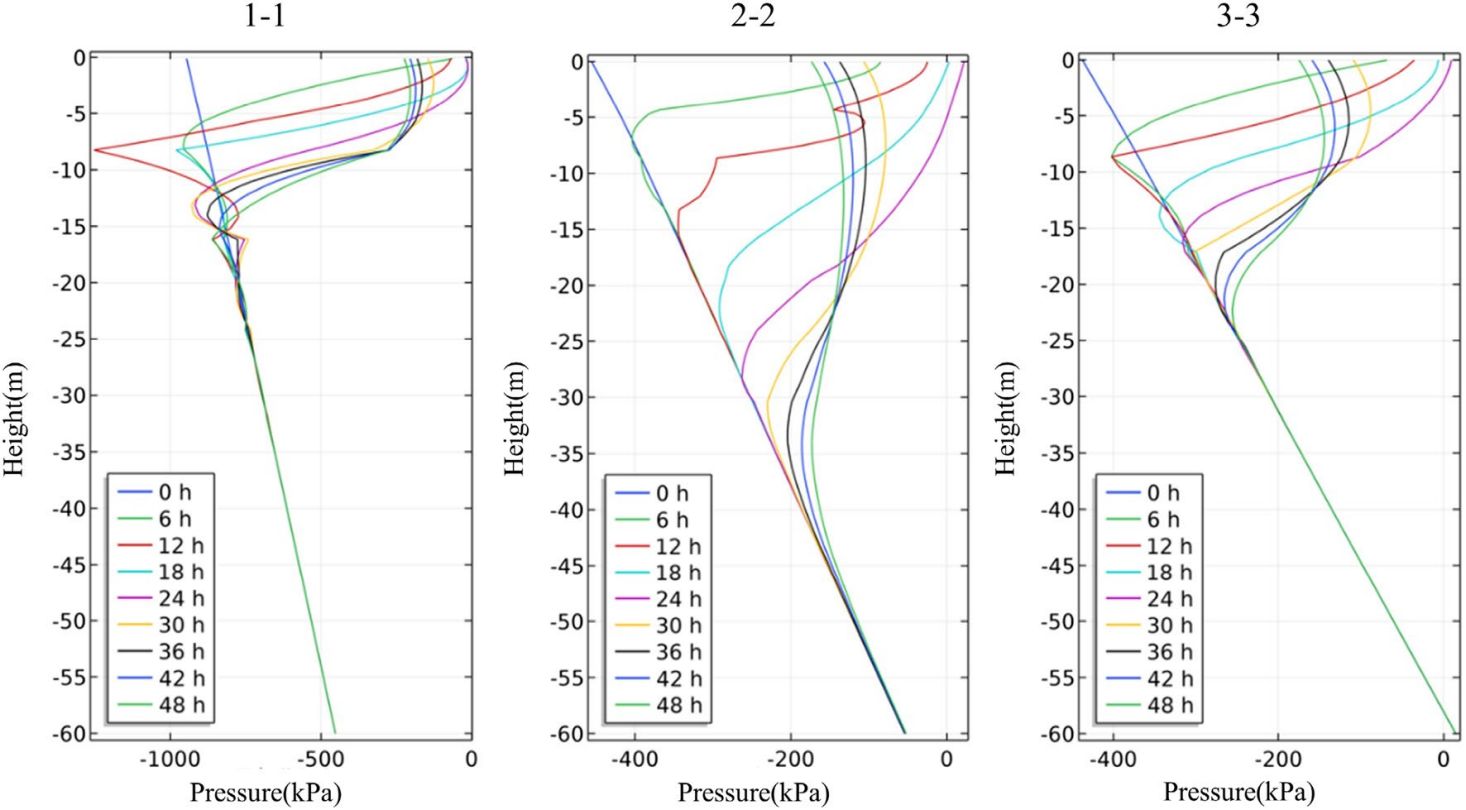
The variation of pore-water pressure in characteristic profiles (Scheme I).

**Figure 19:**
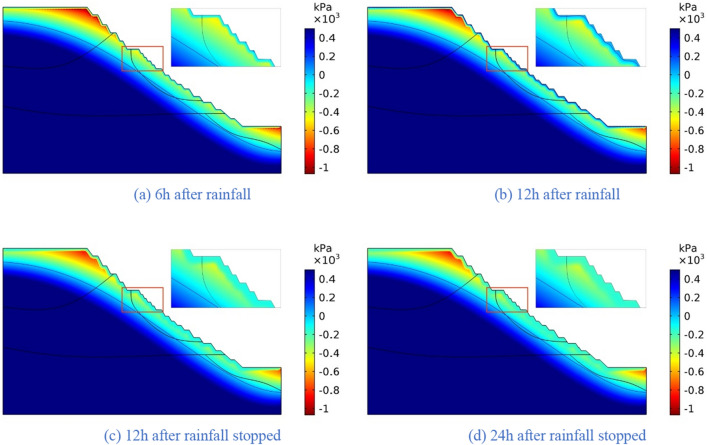
The pore-water pressure distribution (Scheme III).

**Figure 20:**
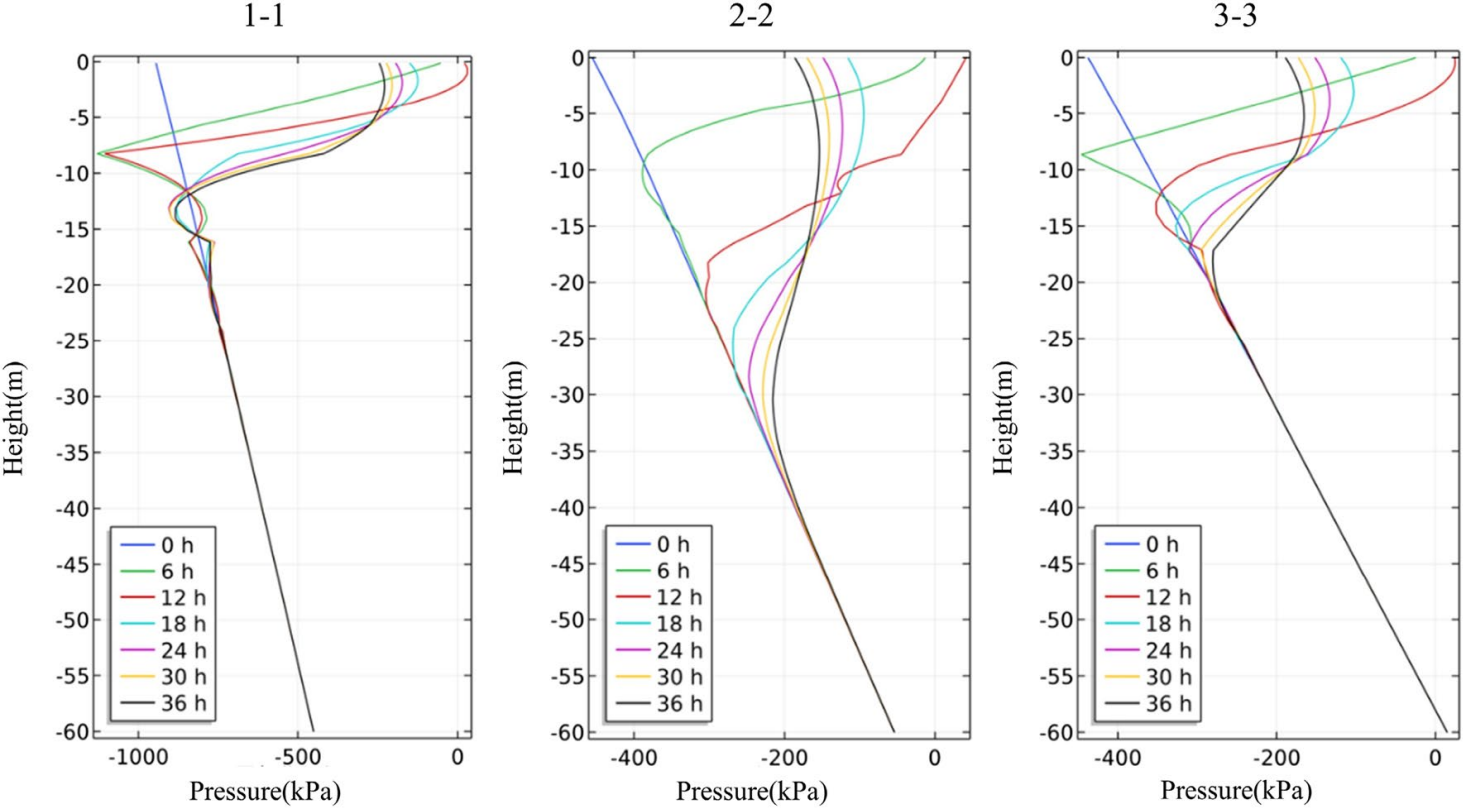
The variation of pore-water pressure in characteristic profiles(Scheme III).

**Figure 21:**
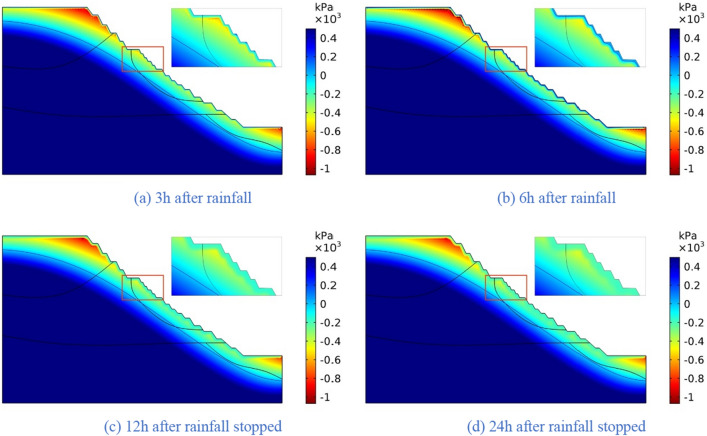
The pore-water pressure distribution (Scheme IV).

**Figure 22:**
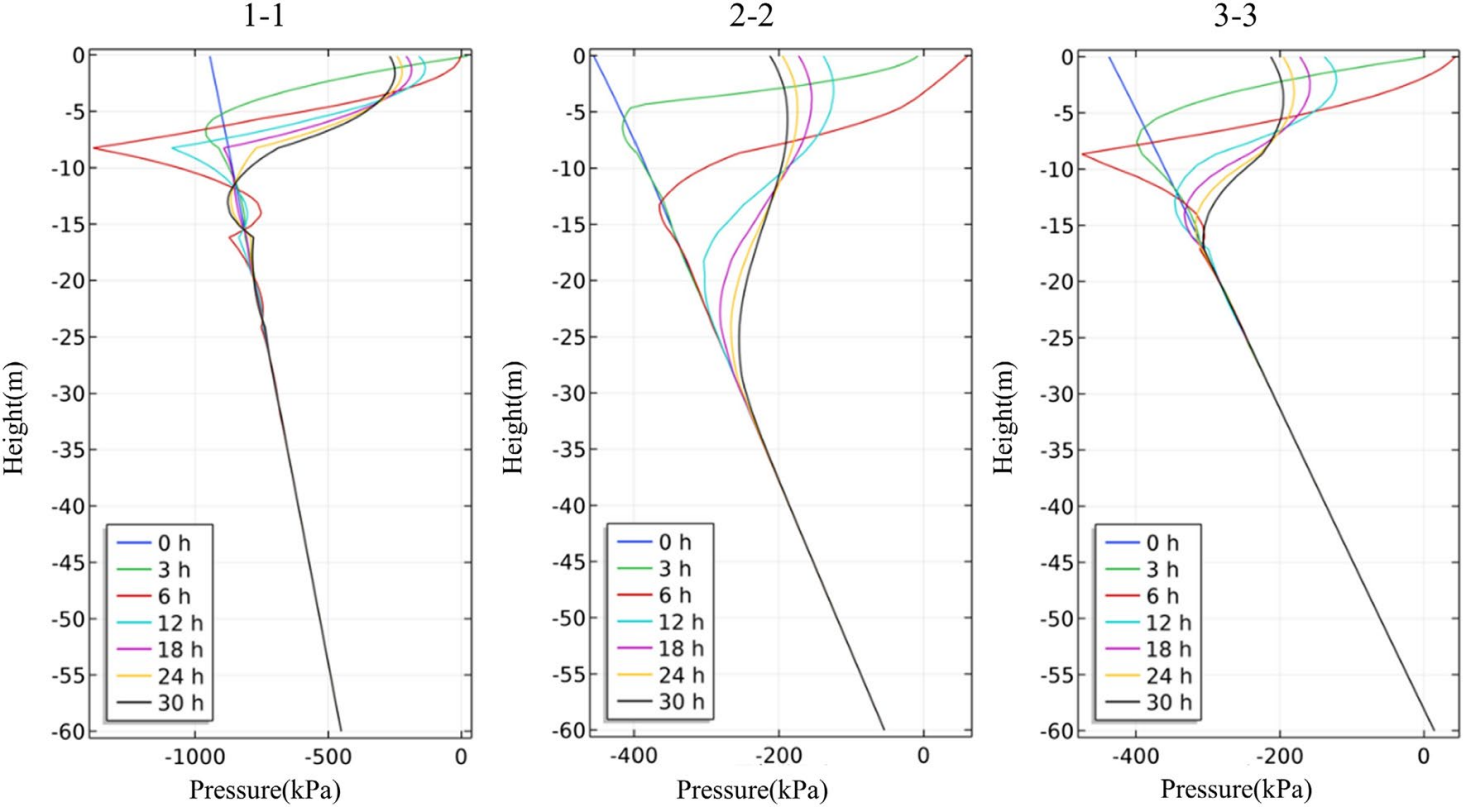
The variation of pore-water pressure in characteristic profiles(Scheme IV).

The four rainfall schemes above are numerically simulated by COMSOL Multiphysics. Within the solution area, 1–1 characteristic profile, 2–2 characteristic profile and 3–3 characteristic profile are selected at the top, middle and bottom of high rock slope for analysis, and the locations of characteristic profiles are shown in Fig. 14. And the calculation results are shown in Fig. 15, 16, 17, 18, 19, 20, 21, 22.

The simulation results of the four rainfall schemes show that: (1) Under continuous rainfall, unsaturated zone on the surface layer of slope gradually evolves into a transient saturated zone and a positive pore-water pressure appears. When the rainfall intensity is constant, the longer the rainfall time is, the larger the transient saturation zone and the hydraulic conductivity depth is. (2) During rainfall, the pore-water pressure of the surface layer of slope gradually increases, and the wetting front evolves to the deeper part. After the rainfall stops, the pore-water pressure gradually decreases in the area above a certain depth; in the area below this depth, the pore-water pressure gradually increases, and the wetting front continues to evolve to a deeper part. (3) When the total rainfall is constant, the greater the rainfall intensity, the greater the growth rate of pore-water pressure in the surface layer of slope, and the greater the maximum pore-water pressure. The longer the rainfall duration, the greater the hydraulic conduction depth. After the rainfall stops, the increase of hydraulic conduction depth is basically the same. (4) During the calculation time, the rainfall infiltration has little effect on the groundwater level because the hydraulic conductivity has not reached the water table surface of groundwater.

## Stability analysis of high rock slope under rainfall infiltration

**Figure 23:**
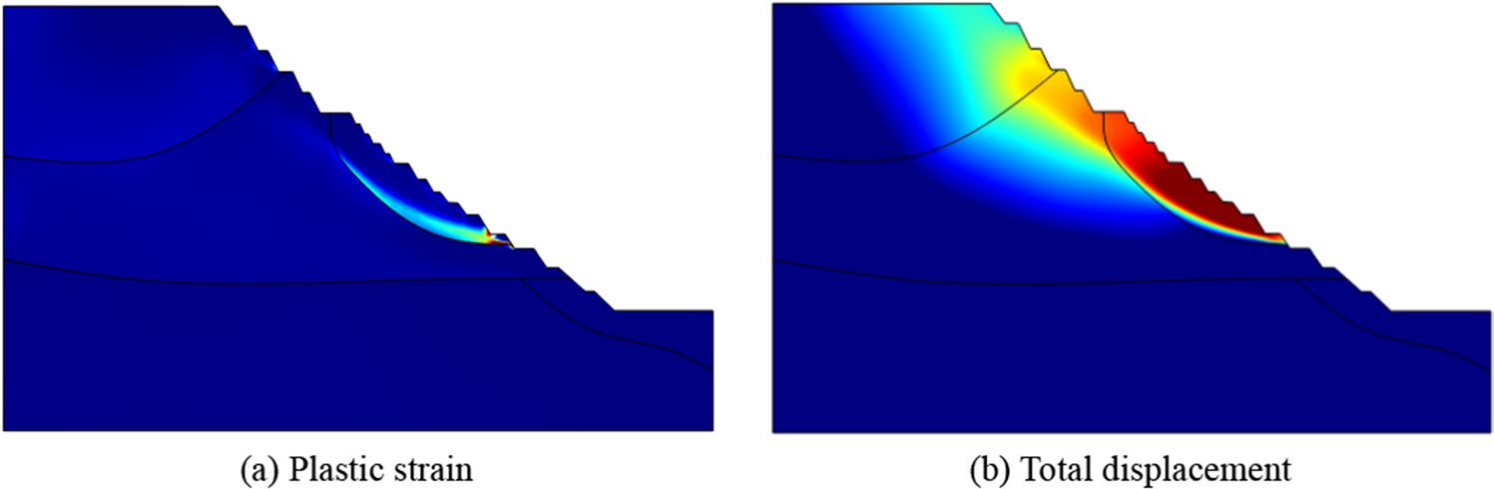
Distribution of plastic strain and total displacement at the initial time.

The proposed nonlinear strength reduction method based on Hoek Brown criterion is used to calculate the safety factor of slope. The minimum principal stress in the slope is calculated so that the cohesion and internal friction angle of the slope rock mass can be obtained. The pore water pressure of slope at the initial time of rainfall (0 h) is substituted into the calculation model, and the strength reduction calculation is carried out for the cohesion and internal friction angle calculated above. Based on the non convergence of calculation, the factor of safety (FOS) at the initial time of rainfall is calculated to be 1.544. The distribution of plastic strain and total displacement at the initial time are shown in Fig. 23.

### Influence of rainfall duration on slope stability

**Figure 24:**
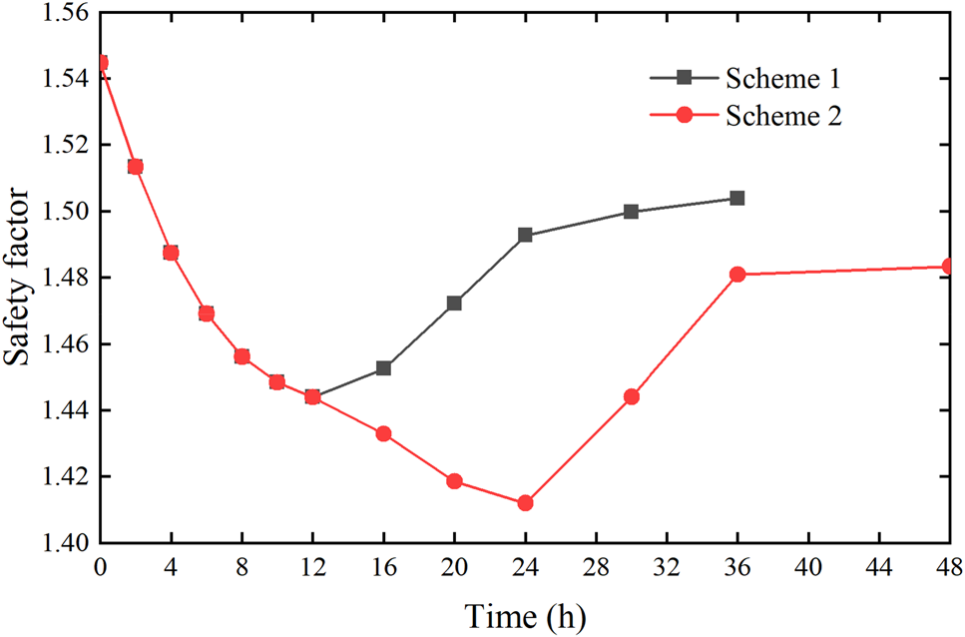
Slope safety factor with time (Scheme I and Scheme II).

The rainfall intensity of Scheme I and Scheme II is 10 mm/h. The rainfall duration of Scheme I is 12 h, the rainfall duration of Scheme II is 24 h, and the calculated duration after the rainfall stops is 24 h. According to the calculation results of the slope safety factor of Scheme I and Scheme II, the change diagram of the slope safety factor with time can be obtained, as shown in Fig. 24.

It can be seen from Fig. 24 that the safety factor of the slope is 1.544 at the beginning of the rainfall. When the rainfall intensity remains unchanged, the safety factor decreases gradually with the increase of rainfall duration. The safety factor is 1.443 and decreases by 0.101 in 12 h of rainfall. The rainfall of Scheme I stops at this time. Then the safety factor starts to rise gradually, and it rises to 1.504 at 24 h after stopping the rainfall. This is because the increment of water load is large, the pore water pressure is increasing and the matrix suction is decreasing when it rains. It leads the safety factor of slope to decrease. When the rainfall stops, there is no rainwater supply, and the previously infiltrated rainwater continues to infiltrate under the effect of gravity. Then the pore water pressure on the upper part of the slope surface gradually decreases. It leads the safety factor to increase. But because the dissipation speed of pore water pressure is relatively slow, the safety factor gradually tends to be stable but less than the safety factor at the initial time.

In Scheme II, the rainfall continues for 24 h and the safety factor decreases to 1.412 at 24 h after raining. After 16 h, the decrease rate of safety factor increases, which is due to the formation of transient saturated zone (TSZ) on the surface of the slope. TSZ has a great impact on the stability of the slope. After the rainfall stops, the safety factor of Scheme II rises gradually to 1.483 at 24 h which is smaller than the safety factor of Scheme I at 24 h after rain.

### Influence of rainfall intensity on slope stability

1. The same rainfall duration

**Figure 25:**
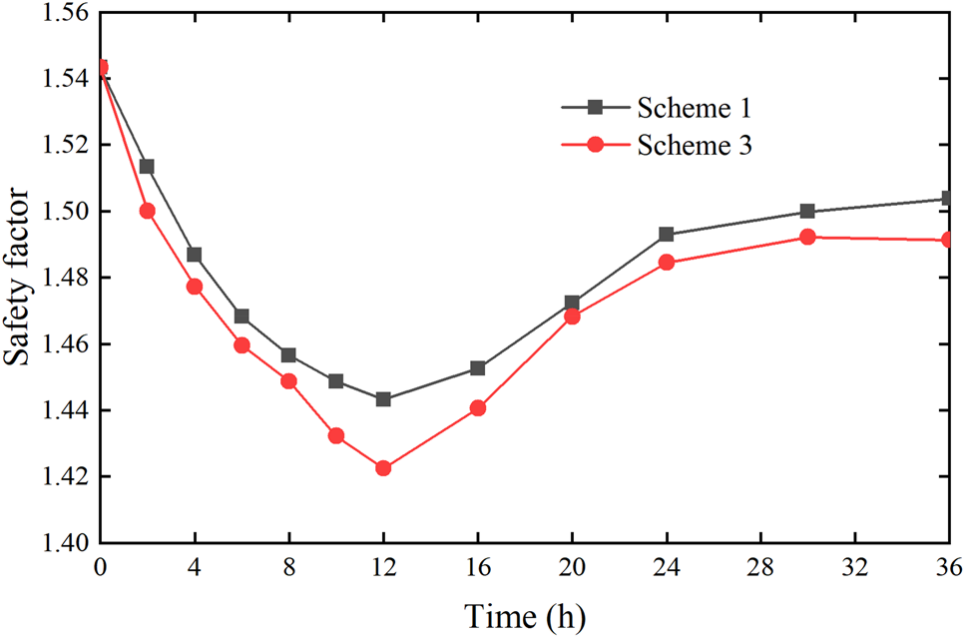
Slope safety factor with time (Scheme I and Scheme III).

The rainfall intensity of Scheme I is 10 mm/h, and that of Scheme II is 20 mm/h. The rainfall duration of the two schemes is the same, both of them are 12 h, and the calculated time length after the rainfall stops is 24 h. According to the calculation results of the slope safety factor of Scheme I and Scheme III, the change diagram of the slope safety factor with time can be obtained, as shown in Fig. 25.

It can be seen from Fig. 25 that the changes in safety factor of Scheme I and Scheme III are similar, both of them reach the lowest level in 12 h of continuous rainfall, and the safety factor of Scheme III is lower than that Scheme I. In the meantime, in the duration of rainfall and the time after the rainfall stopped, the safety factor of Scheme III is always lower than that of Scheme I. For Scheme III, the decrease rate of safety factor increases after 8 h, mainly because the formation of TSZ has a greater impact on slope stability. Overall, the comparison of Scheme I and Scheme III shows that when the rainfall duration is the same, the greater the rainfall intensity is, the lower the slope safety factor is

(2) The same total rainfall

**Figure 26:**
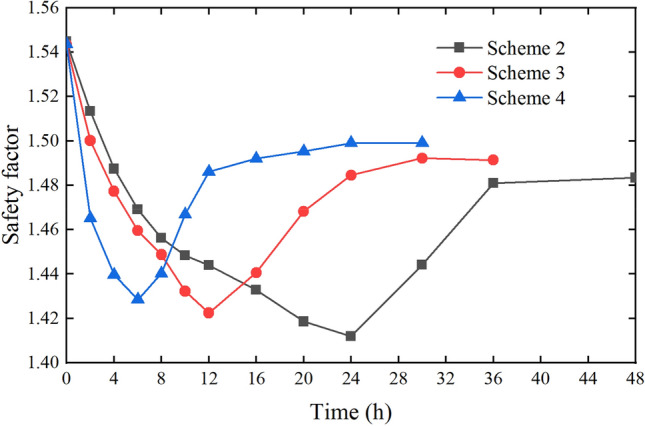
Slope safety factor with time (Scheme II, Scheme III and Scheme IV).

The total rainfall of Scheme II, Scheme III and Scheme IV is 240 mm, the rainfall intensity is respectively 10 mm/h, 20 mm/h and 40 mm/h, the duration is respectively 24 h, 12 h and 6 h, and the calculated duration after the rainfall stops is 24 h. According to the calculation results of the slope safety factor of Scheme II, Scheme III and Scheme IV, the change diagram of the slope safety factor with time can be obtained, as shown in Fig. 26.

It can be seen from Fig. 26 that the safety factors of the three schemes gradually decrease with the continuous rainfall. All of the safety factors become the smallest at the end of the rainfall, and then they gradually recover and increase in a period of time after the rainfall stops. In the process of continuous rainfall, the safety factor of Scheme IV decreases the fastest, followed by Scheme III. This is due to the large rainfall in a short time, the large increment of water load in the slope and the large change of safety factor. Although the total rainfall of the three schemes is equal, the minimum safety factors of the three schemes are different. The minimum safety factor of Scheme IV is the largest, Scheme III is the second, and Scheme II is the smallest. When the rainfall stops (24 h), the safety factor of Scheme 4 is also the largest among the three schemes. The main reason for this phenomenon is that for heavy rainfall in a short time, although the water load increment is large, due to the short infiltration time of rainwater into the slope, the shallow hydraulic conduction depth, and some rainwater will form surface runoff. Although the rainfall intensity for a long time is smaller, the rainwater has sufficient time to continuously infiltrate, the infiltration depth into the slope is greater, the area of rainfall infiltration is larger, and the impact on slope stability is greater. The comprehensive analysis of the three schemes shows that when the total rainfall is the same, the impact of short-term heavy rainfall on slope stability is less than long-term ordinary rainfall, that is, the longer the rainfall duration, the greater the impact on slope stability.

**Figure 27:**
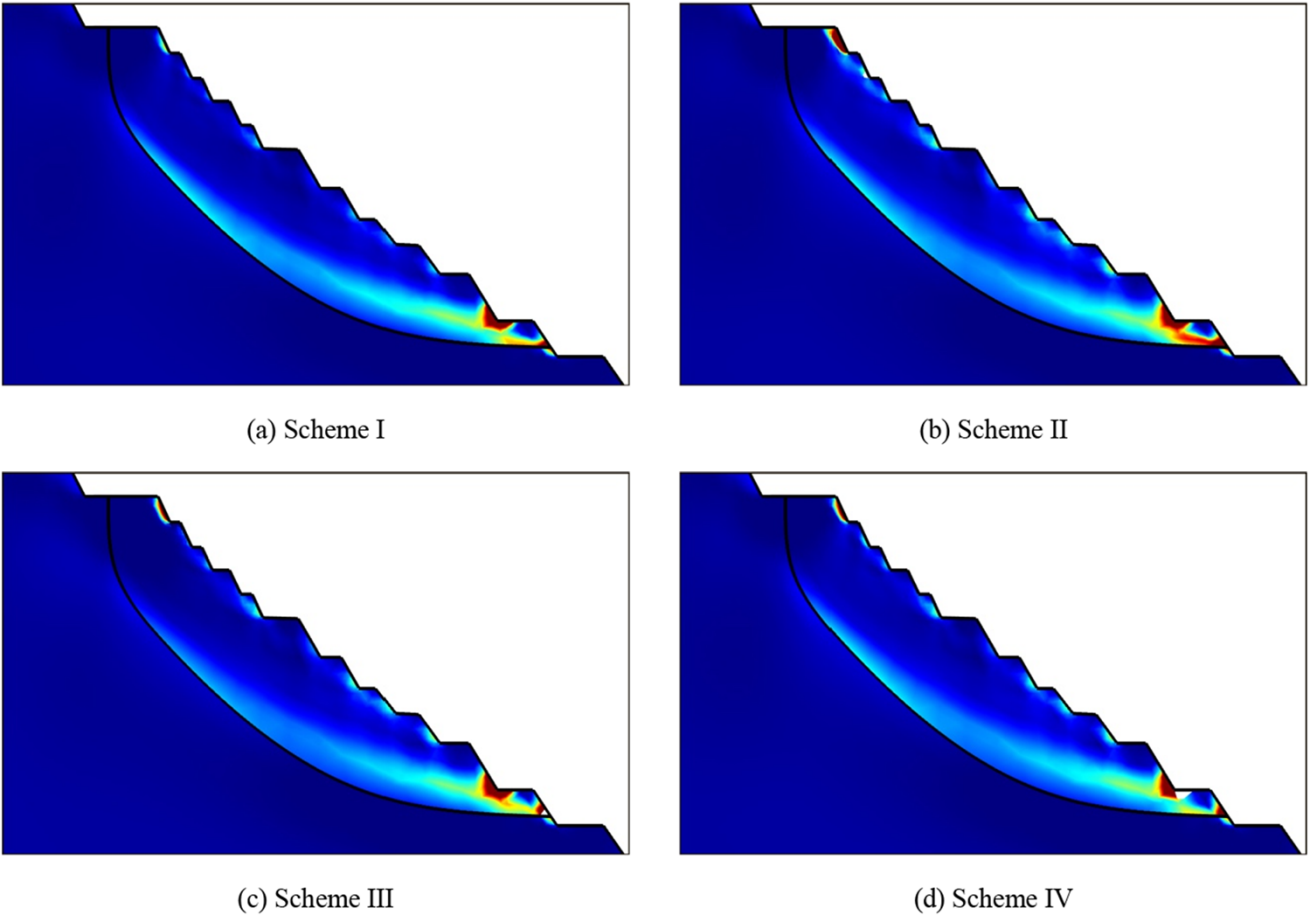
Distribution of plastic strain at the end of rainfall.

At the end of rainfall, the plastic strain distribution of slope are shown in Fig. 27. In the figure, (a), (b), (c) and (d) represent the plastic strain of Scheme I at 12 h, Scheme II at 24 h, Scheme III at 12 h and Scheme IV at 6 h respectively. Comparing Fig. 23 with Fig. 27, it can be seen that the overall plastic zone does not change much, but there is a through plastic zone on some single steps of the slope, especially in Scheme II, which shows that the impact of rainfall infiltration on the stability of some single steps is greater than that of the overall slope. In the process of rainfall, the pore water pressure on the slope surface increases continuously, forming a transient saturation zone, and the rock mass of some steps reaches a transient saturation state. At this time, the water content of rock mass increases, the shear strength decreases, and the sliding force increases, resulting in a significant reduction in the stability of some single steps. This situation often occurs in practical engineering, and long-term rainfall could cause the deformation and failure of a single step or several adjacent steps of the slope.

As mentioned above, a comparative analysis of the four schemes reveals that the FOS will decrease with the increase of rainfall duration. Under the same rainfall duration, the higher the rainfall intensity, the lower the FOS of slope. And under the same total rainfall, the effect of short-term rainstorm on slope stability is smaller than that of long-term ordinary rainfall, and the longer the duration of rainfall, the greater the impact on slope stability.

## Discussion

The high rock slope stability analysis of Dagushan Mine under rainfall infiltration is studied and simulated by the proposed nonlinear strength reduction method. By comparing with the equivalent Mohr- Coulomb method, it is found that high rock slope stability could be more accurately calculated by the proposed method due to its full consideration of slope stress state effect. The seepage characteristics and stability of high rock slope under rainfall infiltration are simulated, and it is found that the effect of short-term heavy rainfall on slope stability is less than that of long-term ordinary rainfall under the same rainfall amount. Model applicability to stability studiy of high rock slope in open-pit mine is confirmed, and its simulation in high rock slope should be applied more widely. In this paper, the equivalent continuous medium model is used as the seepage model. However, the actual fracture yield, spacing and size are randomly distributed. It is necessary to determine the permeability tensor according to the fracture distribution characteristics to make the calculation more realistic. The numerical model is selected as a two-dimensional characteristic profile, while the geometry and rock structures of slope in engineering are anisotropic. The main limitation of the model is that it is only applicable to two-dimensional conditions. It is necessary to optimize the proposed model so that it can be applied to the analysis under three-dimensional conditions in further research.

## Conclusion

1. According to the characteristics of high rock slope with large stress variation range, the instantaneous cohesion and internal friction angle of each slope element under different minimum principal stresses are derived based on Hoek–Brown strength criterion by considering the influence of slope element stress state. On this basis, the nonlinear strength reduction method for high rock slope is proposed.
2. By comparing with the equivalent Mohr- Coulomb method, it is found that the proposed method calculates a smaller landslide body and the potential sliding surface of slope is closer to slope surface. When the slope is low, the difference between the calculation results of the equivalent Mohr- Coulomb method and the proposed method is small, but with the increase of the slope height, the difference between the two calculation results gradually increases.
3. Under continuous rainfall, the pore- water pressure gradually rises and the matrix suction gradually decreases, causing the FOS to gradually decrease. When the rainfall stops, the pore- water pressure in the upper of slope surface layer gradually decreases, and FOS gradually increases but is smaller than the initial FOS. The transient saturated zone has a greater influence on the slope stability. When the transient saturated is formed in the slope surface layer and gradually increases, the reduction rate of FOS gradually increases.
4. Keeping the rainfall intensity, the greater the rainfall duration, the smaller the FOS; keeping the rainfall duration, the greater the rainfall intensity, the smaller the FOS. When the total rainfall is the same, the effect of short-term heavy rainfall on slope stability is less than that of long-term ordinary rainfall.
5. The proposed model could be widely used in the stability analysis of high rock slope, transient saturated zone and long-term ordinary rainfall should be treated as important concerns in policy implications of slope stability analysis. However, the main limitation is that it is not applicable to three-dimensional condition and simplifies the calculation of infiltration tensor, more simulation studies will be presented in our further work.

## Data Availability

The data that support the findings of this study are available from the corresponding author upon reasonable request.

## References

[1] Huang R (2008). Geodynamical process and stability control of high rock slope development. Yanshilixue Yu Gongcheng Xuebao/Chin. J. Rock Mech. Eng..

[2] Sarma S (1979). Stability analysis of embankments and slope. J. Geotech. Eng..

[3] Chen Z, Morgenstern N (2011). Extensions to the generalized method of slices for stability analysis. Can. Geotech. J..

[4] Chen Z, Mi H, Wang X (2001). Limit equilibrium method for three-dimensional analysis of slope stability. Chin. J. Geotech. Eng..

[5] Zou J, David J (1995). Search for critical slip surface based on finite element method. Can. Geotech. J..

[6] Duncan J (1996). State of the art: Limit equilibrium and finite-element analysis of slopes. J. Geotech. Eng..

[7] Xu J, Pan Q, Yang X (2018). Stability charts for rock slopes subjected to water drawdown based on the modified nonlinear Hoek-Brown failure criterion. Int. J. Geomech..

[8] Wang H, Yang Y, Sun G (2021). A stability analysis of rock slopes using a nonlinear strength reduction numerical manifold method. Comput. Geotech..

[9] Rezaur R, Rahardjo H, Leong E (2002). Spatial and temporal variability of pore-water pressures in residual soil slopes in a tropical climate. Earth Surf. Proc. Land..

[10] Alonso E, Gens A, Delahaye C (2003). Influence of rainfall on the deformation and stability of a slope in overconsolidated clays: A case study. Hydrogeol. J..

[11] Verma A, Singh T, Chauhan N (2016). A hybrid FEM–ANN approach for slope instability prediction. J. Inst. Eng. (India) Ser. A.

[12] Rahimi A, Rahardjo H, Leong E (2010). Effect of hydraulic properties of soil on rainfall-induced slope failure. Eng. Geol..

[13] McQuillan A, Canbulat I, Oh J (2020). Methods applied in Australian industry to evaluate coal mine slope stability. Int. J. Min. Sci. Technol..

[14] Hoek E, Brown E (1980). Empirical strength criterion for rock masses. J. Geotech. Eng. Div..

[15] Fu W, Yi L (2010). Non-linear shear strength reduction technique in slope stability calculation. Comput. Geotech..

[16] Zhao L (2010). Seismic stability quasi-static analysis of homogeneous rock slopes with Hoek-Brown failure criterion. Chin. Civil Eng. J..

[17] Chowdhury R (1980). Slope analysis. Developments in geotechnical engineering. Int. J. Rock Mech. Mini. Sci. Geomech..

[18] Shen J, Karakus M, Xu C (2012). Direct expressions for linearization of shear strength envelopes given by the Generalized Hoek-Brown criterion using genetic programming. Comput. Geotech..

[19] Meng Q, Wang H, Xu W (2019). Multiscale strength reduction method for heterogeneous slope using hierarchical FEM/DEM modeling. Comput. Geotech..

[20] Liao Y, Zeng X, Fu W (2012). Linearization method of non-linear strength of Hoek-Brown rock mass. Zhongnan Daxue Xuebao (Ziran Kexue Ban)/J. Central South Univ. (Sci. Technol.).

[21] Yuan W, Li J, Li Z (2020). A strength reduction method based on the Generalized Hoek-Brown (GHB) criterion for rock slope stability analysis. Comput. Geotech..

[22] Saada Z, Maghous S, Garnier D (2011). Seismic bearing capacity of shallow foundations near rock slopes using the generalized Hoek-Brown criterion. Int. J. Numer. Anal. Meth. Geomech..

[23] Wu S, Jin A, Gao Y (2006). Numerical simulation analysis on strength reduction for slope of jointed rock masses based on gereralized Hoek-Brown failure criterion. Chin. J. Geotech. Eng..

[24] Kumar N, Verma A, Sardana S (2018). Comparative analysis of limit equilibrium and numerical methods for prediction of a landslide. Bull. Eng. Geol. Environ..

[25] Azarafza M, Akgün H, Ghazifard A (2021). Discontinuous rock slope stability analysis by limit equilibrium approaches-a review. Int. J. Digital Earth.

[26] Yuan W, Li X, Wang W (2016). Study on strength reduction method based on double reduction parameters. Rock Soil Mech..

[27] Nekouei A, Ahangari K (2013). Validation of Hoek-Brown failure criterion charts for rock slopes. Int. J. Min. Sci. Technol..

[28] Lin H, Cao P, Li J (2008). The indirect calculation method for the safety factor of slope based on generalized Hoek-Brown criterion. J. China Coal Soc..

[29] Lin H, Cao P, Zhao Y (2007). Application of strength reduction method in Hoek-Brown criterion. J. Central South Univ. Sci. Technol..

[30] Hammah R, Yacoub T, Corkum B, et al. The shear strength reduction method for the generalized Hoek-Brown criterion. Proceedings of the 40th US symposium on rock mechanics, Alaska Rocks 2005, Anchorage, Alaska. 2005.

[31] Thomas B, Radu S, Regina A (2008). A Hoek-Brown criterion with intrinsic material strength factorization. Int. J. Rock Mech. Mini. Sci..

[32] Wu S, Xiong L, Zhang S (2018). Strength reduction method for slope stability analysis based on a dual factoring strategy. Int. J. Geomech..

[33] Li A, Merifield R, Lyamin A (2008). Stability charts for rock slopes based on the Hoek-Brown failure criterion. Int. J. Rock Mech. Min. Sci..

[34] Sun C, Chai J, Xu Z (2016). Stability charts for rock mass slopes based on the Hoek-Brown strength reduction technique. Eng. Geol..

[35] Benz T, Schwab R, Kauther R (2008). A Hoek-Brown criterion with intrinsic material strength factorization. Int. J. Rock Mech. Min. Sci..

[36] Yang X, Yin J (2010). Slope equivalent Mohr-Coulomb strength parameters for rock masses satisfying the Hoek-Brown criterion. Rock Mech. Rock Eng..

[37] Meng Q, Qian K, Zhong L (2020). Numerical analysis of slope stability under reservoir water level fluctuations using a FEM-LEM-Combined method. Geofluids.

[38] Zheng H, Sun G, Liu D (2009). A practical procedure for searching critical slip surfaces of slopes based on the strength reduction technique. Comput. Geotech..

[39] Zhao S, Zheng Y, Deng W (2003). Stability analysis of jointed rock slope by strength reduction method FEM. Chin. J. Rock Mech. Eng..

[40] Zong Q, Xu W (2008). Stability analysis of excavating rock slope using generalized Hoek-Brown failure criterion. Rock Soil Mech..

[41] Song Q, Yan E, Mao W (2012). Determination of Shear Strength Reduction Factor for generalized Hoek-Brown criterion. Chin. J. Rock Mech. Eng..

[42] Zhang L, Yang D, Chen Z (2020). Deformation and failure characteristics of sandstone under uniaxial compression using distributed fiber optic strain sensing. J. Rock Mech. Geotech. Eng..

[43] Zhuo L, He J, Xie H (2015). Study of new method to determine strength parameters of rock material based on hoek-brown criterion. Chin. J. Rock Mech. Eng..

[44] Zhang L, Zhang J, Zhang L (2011). Stability analysis of rainfall-induced slope failure: A review. Proceedings of the Institution of Civil Engineers. Geotech. Eng..

[45] Ma L, Yang F, Wang M (2017). Generalized Hoek-Brown dynamic strength criterion incorporating strain rate effect. Yantu Lixue/Rock Soil Mech..

